# Antioxidant, Structural and Technofunctional Profiling of Seed-Free Sea Buckthorn Pomace Dry Fractionated by Grinding–Sieving

**DOI:** 10.3390/foods15203668

**Published:** 2026-10-15

**Authors:** Andreea Ghitulescu, Vlad Enache, Mariana Suba, Catalin Ianasi, Diana Moigradean, Mariana-Atena Poiana

**Affiliations:** 1 Doctoral School “Engineering of Plant and Animal Resources”, University of Life Sciences “King Mihai I” from Timisoara, Aradului Street No. 119, 300645 Timisoara, Romania; andreea.ghitulescu@usvt.ro (A.G.); vlad.enache@usvt.ro (V.E.); 2 Faculty of Food Engineering, University of Life Sciences “King Mihai I” from Timisoara, Aradului Street No. 119, 300645 Timisoara, Romania; dianamoigradean@usvt.ro; 3 ”Coriolan Dragulescu” Institute of Chemistry, Romanian Academy, Bd. Mihai Viteazul No. 24, 300223 Timisoara, Romania; mariana_suba@acad-icht.tm.edu.ro (M.S.); ianasic@acad-icht.tm.edu.ro (C.I.)

**Keywords:** sea buckthorn pomace, granulometric fractions, particle size distribution, hydration properties, phytochemicals, antioxidant activity, structural characterization

## Abstract

Sea buckthorn pomace, generated as a by-product of juice processing, has potential for valorisation as a functional food ingredient. This study investigated whether dry fractionation by grinding and controlled sieving of seed-free sea buckthorn pomace (SBP) can generate granulometric fractions with distinct compositional, antioxidant, structural, and technofunctional characteristics. SBP was separated into six fractions (SBPF1: >2.0 mm; SBPF2: 1.0–2.0 mm; SBPF3: 0.5–1.0 mm; SBPF4: 0.25–0.5 mm; SBPF5: 0.15–0.25 mm; SBPF6: <0.15 mm) and evaluated for proximate composition, colour, water swelling capacity (WSC), water retention capacity (WRC), water solubility index (WSI), oil retention capacity (ORC), total phenolic content (TPC), total flavonoid content (TFC), DPPH radical-scavenging activity, ferric reducing antioxidant power (FRAP), and structural characteristics. Granulometric fractionation resulted in significant differences among the fractions. SBPF1 showed the highest total dietary fibre content (58.46%), with insoluble dietary fibre predominating in the coarser fractions, whereas soluble dietary fibre increased towards the finer fractions. WSC was highest in SBPF3, WRC in SBPF5, WSI increased progressively towards SBPF6, and ORC was higher in the coarser fractions. SBPF4 showed the highest TPC (2984.02 mg GAE/100 g d.w.), TFC (1365.14 mg QE/100 g d.w.), DPPH (315.74 μmol TE/g d.w.), and FRAP (277.63 μmol Fe^2+^/g d.w.) values. Pearson analysis showed strong positive correlations among TPC, TFC, DPPH, and FRAP, while principal component and agglomerative hierarchical analyses differentiated the granulometric fractions according to their multivariate profiles. Attenuated total reflectance Fourier-transform infrared (ATR-FTIR) spectroscopy showed preservation of the main absorption regions together with fraction-specific differences in spectral parameters and peak-area ratios, whereas wide-angle X-ray scattering (WAXS) profiles remained broadly comparable, with a diffuse feature around a scattering angle (2θ) of 20° and no newly appearing sharp reflections, although crystallite size differed among fractions and increased from SBPF3 to SBPF6. These results demonstrate that granulometric fractionation can generate SBP fractions with distinct characteristics, supporting their consideration as potential value-added ingredients for further evaluation in specific food applications.

## 1. Introduction

The generation of agro-industrial by-products from fruit processing has stimulated sustainable valorization strategies aligned with the circular bioeconomy and zero-waste food systems [1,2]. Fruit pomaces are valuable raw materials rich in dietary fibre, phenolic compounds, carotenoids, vitamins, and other bioactive compounds, offering considerable potential for developing functional food ingredients [3,4]. Among these, sea buckthorn (*Hippophae rhamnoides* L.) pomace from juice processing is a promising source of bioactives and dietary fibre, supporting its valorization as a sustainable multifunctional ingredient [5,6,7]. However, the recovery and concentration of these high-value constituents have relied predominantly on conventional and advanced solvent-based extraction techniques [8,9]. These methods often require complex processing, specialized equipment, and organic solvents, raising concerns regarding environmental impact, operational costs, and the preservation of native matrix functionality [9,10]. Moreover, such techniques isolate bioactive compounds from their natural structural environment, limiting the direct utilization of the intact plant matrix as a multifunctional food ingredient [11].

Direct utilization of powders or granulometric fractions derived from plant materials or agro-industrial by-products represents a sustainable alternative, preserving bioactive compounds within the native matrix and maintaining their natural association with dietary fibre and structural components without requiring further extraction steps [11,12,13].

Dry fractionation by grinding and sieving has emerged as a sustainable alternative to solvent-based extraction, producing compositionally distinct plant fractions while preserving the native association between dietary fibre, phytochemicals and structural constituents. Its low energy requirements, absence of organic solvents and industrial scalability support the development of clean-label functional ingredients [14,15,16,17]. Beyond its environmental advantages, this procedure preserves the integrity of the plant matrix, allowing the simultaneous enrichment of naturally associated nutrients and bioactive constituents within the resulting fractions [14,18,19]. During grinding and controlled differential sieving, plant tissues are disrupted according to differences in hardness, fibrousness, and mechanical resistance, leading to the selective redistribution of different tissue components among the granulometric fractions rather than simple size reduction [20,21,22,23].

Particle size is a major determinant of the physicochemical and functional properties of plant-derived fractions, influencing cell disruption, fibrous structure retention and the distribution of dietary fibre, phytochemicals and antioxidant activity [11,24,25,26,27]. Previous studies have shown that dry fractionation by grinding–sieving modifies the distribution of fibre, phenolics, flavonoids, minerals and antioxidant activity [25,28], although bioactive compounds are not always concentrated in the finest fractions due to tissue redistribution and grinding-induced oxidation [11,18,27]. From a technological perspective, particle size governs the functional behaviour of food powders, influencing processing performance, hydration and solubility characteristics, as well as technological functionality. Sieve-based separation provides a practical means of obtaining well-defined granulometric classes with distinct techno-functional attributes [29]. Fine plant-derived powders below approximately 150 µm have been associated with increased specific surface area, enhanced dissolution of phytochemicals and improved dispersion, characteristics relevant for beverage-type and semi-liquid systems [30]. In addition, particle size has been shown to influence viscosity, turbidity and sedimentation behaviour in carrot pomace-fortified milk drinks [31], as well as particle perception and grittiness in yogurt-type systems containing apple pomace [32]. Intermediate particle-size domains, generally situated between roughly 150 and 500 µm, have been investigated in bakery applications, where particle size influences water and oil interactions, dough behaviour, gluten-network development, texture and product quality [33,34]. Similar particle-size effects have been reported for grape pomace incorporated into muffins [35]. Coarser fibre-rich particles above about 500 µm exhibit distinct hydration and structural behaviour, with documented effects on dough rheology, texture and sensory attributes, as shown for apple pomace fractions extending to 840 µm [36]. These studies demonstrate that different granulometric domains are associated with distinct technological behaviours and application contexts, providing a literature-based framework for selecting particle-size ranges relevant to food applications. For food applications, plant-based fractions with different particle sizes should be evaluated not only for their chemical composition but also for their technofunctional properties. Hydration characteristics such as water retention capacity, swelling capacity and water solubility index are closely linked to particle size, fibre composition and the structural organization of the plant matrix, influencing viscosity development and processing behaviour [25,27,37,38]. Integrated characterization combining composition, phytochemicals, antioxidant activity and technofunctional properties is therefore essential for evaluating the functional potential of granulometric fractions and supporting their selection for specific food applications [11,28,39]. However, these analyses provide limited insight into the structural characteristics associated with grinding and sieving [28]. Complementary structural characterization can therefore provide additional information on the organization of the plant matrix and assist in interpreting the functional differences among granulometric fractions. ATR-FTIR spectroscopy enables the identification of functional groups associated with polysaccharides, lignocellulosic components, proteins and phenolic compounds, allowing comparison of the spectral characteristics of different granulometric fractions. Meanwhile, wide-angle X-ray scattering (WAXS) offers supplementary information regarding molecular ordering and scattering characteristics within the plant matrix [40]. When combined with compositional, phytochemical and technofunctional analyses, these structural techniques provide complementary information that helps interpret the differences observed among granulometric fractions.

Most studies have investigated whole sea buckthorn pomace, although this by-product contains peel, pulp, and seeds with distinct compositions and structural characteristics [5,41]. Because dry fractionation by a grinding–sieving procedure separates tissues according to their mechanical behaviour during grinding and sieving [11,12,22], the presence of seeds may introduce additional variability among granulometric fractions. Consequently, removing the seeds prior to processing enables a more accurate assessment of the redistribution of peel–pulp constituents among granulometric fractions and their contribution to the compositional, antioxidant, and technofunctional characteristics of the resulting materials [11,12]. To investigate this redistribution, the present study focused on seed-free sea buckthorn pomace. Prior research on sea buckthorn pomace has primarily focused on phytochemical profiling, antioxidant and biological activities, and the extraction and recovery of individual bioactive compounds from whole pomace [5,8,9]. However, dry fractionation of sea buckthorn pomace and the comprehensive characterization of the resulting granulometric fractions remain unexplored, particularly regarding their compositional, phytochemical, technofunctional, and structural characteristics.

To address this gap, the study hypothesized that dry fractionation by grinding and controlled sieving of seed-free sea buckthorn pomace can generate granulometric fractions with distinct compositional, antioxidant, structural, and technofunctional characteristics. The resulting fractions were evaluated for proximate composition, phytochemical profile, antioxidant activity, colour, hydration and oil-retention properties, and structural characteristics using ATR-FTIR spectroscopy and WAXS. The study aimed to support the selection of granulometric fractions with potential use as sustainable functional food ingredients and to provide a basis for their further evaluation in specific food applications.

## 2. Materials and Methods

### 2.1. Chemicals and Analytical Reagents

All chemicals and reagents used in this study were of analytical grade. Folin–Ciocalteu reagent, anhydrous sodium carbonate, ferric chloride hexahydrate, sodium hydroxide, ethanol (96%), glacial acetic acid, and hydrochloric acid (0.1 M) were purchased from Merck (Darmstadt, Germany). Gallic acid, quercetin, 2,2-diphenyl-1-picrylhydrazyl (DPPH), 2,4,6-tris(2-pyridyl)-s-triazine (TPTZ), Trolox (6-hydroxy-2,5,7,8-tetramethylchroman-2-carboxylic acid), ferrous sulfate heptahydrate, anhydrous sodium acetate, sodium nitrite, and aluminum nitrate nonahydrate were obtained from Sigma-Aldrich (Taufkirchen, Germany).

### 2.2. Plant Material

**Figure 1:**
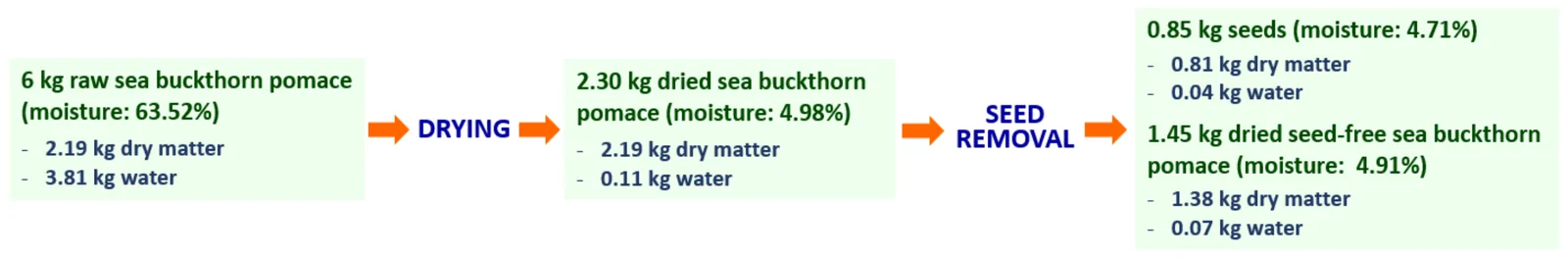
Mass and moisture distribution of sea buckthorn pomace during drying and seed removal.

The raw sea buckthorn pomace (*Hippophae rhamnoides* L.), consisting of pulp, peel, and seeds, was obtained as a by-product of cold-pressed sea buckthorn juice production from Din Eden Produse Naturale S.R.L. (Polovragi, Gorj County, Romania; 45.167970° N, 23.797116° E) in November 2025. The pomace used in the present study was collected from the bulk by-product generated during a single production batch. A 6.00 kg portion of raw pomace was used for experimental processing. The material was immediately frozen after collection and stored at −22 °C until further processing. The frozen pomace was thawed at 4 °C for 24 h under controlled refrigerated conditions [42]. The thawed pomace, with a moisture content of 63.52%, was subsequently spread in thin, uniform layers on stainless-steel trays and dried in a forced-air convection oven (FM Industrial S.A., Lucena, Córdoba, Spain) at 60 °C for two consecutive days (9 h on the first day and 8 h on the second day) under continuous ventilation. These moderate drying conditions were selected to ensure effective moisture removal while limiting the degradation of phenolic compounds and the loss of antioxidant properties [43,44,45]. Following drying, 2.30 kg of dried pomace was obtained, with a residual moisture content of 4.98%. This mass comprised approximately 2.19 kg of dry matter and 0.11 kg of residual water. After cooling to room temperature, the dried pomace was mechanically separated into seeds and seed-free pomace, yielding 0.85 kg of seeds and 1.45 kg of dried seed-free pomace. Seed separation was performed after drying because, in the fresh pomace, the seeds were embedded in the moist and compact pulp–peel matrix, making their mechanical removal difficult. The resulting dried seed-free pomace had a moisture content of 4.91% and constituted the bulk material used for the subsequent grinding and dry-fractionation experiments. The corresponding mass and moisture distribution throughout drying and seed removal is presented in Figure 1.

### 2.3. Dry Granulometric Fractionation of Seed-Free Sea Buckthorn Pomace by Grinding and Sieving

Dry granulometric fractionation based on grinding followed by sieving was independently performed in triplicate using three separate 200 g experimental samples taken from the dried seed-free sea buckthorn pomace. Each sample was ground using a Robot Coupe Blixer 3 laboratory mill (Robot Coupe, Montceau-les-Mines, France), operating at a constant rotational speed of 1750 rpm. Each 200 g batch was ground in three 20 s cycles, separated by 30 s pauses, resulting in a total effective grinding time of 60 s. The grinding conditions were established through preliminary trials with seed-free sea buckthorn pomace and considered in relation to previously reported grinding and particle-size fractionation procedures for plant-derived materials [19,21,25,39], with the aim of providing sufficient disintegration for subsequent sieving while avoiding excessive particle size reduction. The pauses between successive grinding cycles reduced continuous mechanical exposure and were intended to limit potential heat accumulation.

After grinding, the material from each experimental replicate was thoroughly homogenized and divided into two equal 100 g subsamples. One of them was retained as the unfractionated reference sample (SBP), whereas the other was subjected to the sieving step of the dry-fractionation procedure. Thus, three independently processed SBP samples and three corresponding sets of granulometric fractions were obtained.

Vibratory sieving was performed using a Digital Electromagnetic Sieve Shaker IRIS FTL-0200 (Filtra Vibración, Badalona, Barcelona, Spain). The sieve stack consisted of stainless-steel sieves with mesh openings of 2.0, 1.0, 0.5, 0.25, and 0.15 mm, followed by a collection pan for particles smaller than 0.15 mm. The sieve shaker was operated by progressively increasing the power from levels 5 to 9, with each level maintained for 2 min, resulting in a total processing time of 10 min. The 10 min sieving duration was selected based on procedures previously reported for the granulometric separation of plant-derived fractions [11,19,22,23,25], whereas the stepwise increase in instrument power was established through preliminary trials conducted with sea buckthorn pomace to achieve effective and reproducible separation of the material through the sieve stack. Under these conditions, six distinct granulometric fractions were obtained: SBPF1 (>2.0 mm), SBPF2 (1.0–2.0 mm), SBPF3 (0.5–1.0 mm), SBPF4 (0.25–0.5 mm), SBPF5 (0.15–0.25 mm), and SBPF6 (<0.15 mm). Before each fractionation run, the individual sieves were weighed to determine their tare masses. Following sieving, the sieves containing the retained fractions were reweighed, and the mass of each granulometric fraction was calculated as the difference between the loaded and tare sieve masses. Each fraction obtained from the three independent fractionation runs was maintained separately throughout subsequent analyses and was not pooled across replicates. The resulting fractions and the corresponding unfractionated SBP samples were vacuum-sealed in polyethylene bags and stored at 10 °C until physicochemical, phytochemical, structural, and technofunctional analyses [25].

### 2.4. Particle Size Distribution and Granulometric Parameters

The particle-size distribution of the ground seed-free sea buckthorn pomace was evaluated separately for each of the three independent fractionation experiments based on the mass retained on each sieve after successive sieving [25]. For each experiment, the mass of material retained in each particle-size fraction was recorded individually. Based on these measurements and the corresponding sieve openings (l_i_), the following granulometric parameters were calculated: the average particle size of each fraction (d_i_), the per-centage of material within each particle-size class ($a_{i}$), the cumulative percentage of material with particle sizes smaller than the corresponding sieve opening ($T_{i}$), and the cumulative percentage of material with particle sizes larger than the corresponding sieve opening ($R_{i}$) [25,38]. The average particle size of each fraction ($d_{i}$) was calculated as the arithmetic mean of the openings of two adjacent sieves, according to Equation (1) [25]:

(1) $$d_{i}\;(mm)=\frac{l_{i}+l_{i+1}}{2}$$

where: $l_{i}$ and $l_{i+1}$ are the openings of the two adjacent sieves, expressed in mm.

For the coarsest fraction (>2 mm), the upper sieve opening was estimated assuming that successive sieve openings followed a geometric progression with a ratio of $\sqrt{2}$, as calculated using Equation (2) [25,29].

(2) $$l_{i+1}\;(mm)=l_{i}\times \sqrt{2}$$

The average particle size of the coarsest fraction was subsequently calculated according to Equation (3) [25]:

(3) $$d_{i}\;(mm)=\frac{l_{i}+l_{i}\times \sqrt{2}}{2}$$

The percentage of material within each particle-size class ($a_{i}$), corresponding to the percentage of material with particle sizes between two adjacent sieve openings, was calculated as the ratio between the mass (g) retained in the respective fraction ($m_{i}$) and the total mass (g) of the fractionated sample ($m_{total}$), according to Equation (4) [25]:

(4) $$a_{i}\;(\%)=\frac{m_{i}}{m_{total}}\times 100$$

The cumulative percentages of material with particle sizes smaller ($T_{i}$) and larger ($R_{i}$) than the corresponding sieve opening were calculated by summing the percentages of all particle-size classes smaller and larger than that opening, respectively [25].

### 2.5. Determination of Technofunctional Properties

Water swelling capacity (WSC), water retention capacity (WRC), water solubility index (WSI) and oil retention capacity (ORC) were determined according to the method described by Kaminskytė et al. [46], with minor modifications. For WSC determination, 0.200 g of each sample was transferred into a pre-weighed centrifuge tube. The initial volume of the dry sample ($V_{0}$) was recorded, followed by the addition of 6 mL of distilled water. The samples were gently mixed and allowed to hydrate at 21 °C for 24 h. After hydration, the final volume of the swollen sample ($V_{1}$) was measured, and WSC was calculated according to Equation (5):

(5) $$WSC\;(mL/g)=\frac{\left(V_{1}-V_{0}\right)}{M}$$

where $V_{0}$ represents the initial volume of the dry sample (mL), $V_{1}$ represents the volume of the hydrated sample (mL), and M is the dry mass of the sample (g).

Following WSC determination, the hydrated samples were centrifuged at 5000 rpm for 10 min using a Hettich centrifuge (Andreas Hettich GmbH, Tuttlingen, Germany). The supernatant was carefully removed, and the remaining hydrated residue was weighed ($M_{1}$). The residue was subsequently dried at 105 °C until constant mass was achieved, and the final mass ($M_{2}$) was recorded. WRC was calculated according to Equation (6):

(6) $$WRC\;(g/g)=\frac{\left(M_{1}-M_{2}\right)}{M_{2}}$$

where $M_{1}$ represents the mass of the hydrated residue after centrifugation (g), and $M_{2}$ represents the dry mass of the residue after oven drying (g).

For WSI determination, the collected supernatant was transferred into pre-weighed drying dishes and evaporated at 105 °C until constant mass using a drying oven (AIR Concept, Precisa Gravimetrics AG, Dietikon, Switzerland). The mass of recovered soluble solids ($M_{s}$) was recorded, and WSI was calculated according to Equation (7):

(7) $$WSI\;(\%)=\frac{M_{s}}{M_{0}}\times 100$$

where $M_{s}$ represents the mass of soluble solids recovered after drying the supernatant (g), and $M_{0}$ represents the initial dry mass of the sample used for analysis (g).

Oil retention capacity (ORC) was determined according to Kaminskytė et al. [46]. A 0.200 g sample was mixed with 2.0 g sunflower oil, incubated at 21 °C for 1 h, and centrifuged at 3000 rpm for 10 min. The supernatant was decanted, the residue was weighed, and ORC was calculated according to Equation (8):

(8) $$ORC\;(g/g)=\frac{\left(M_{1}-M_{0}\right)}{M_{0}}\times 100$$

where $M_{1}$ represents the mass of the residue after centrifugation (g), and $M_{0}$ represents the dry mass of the original sample (g).

### 2.6. Proximate Analysis and Energy Value

The proximate composition of SBP and SBPF1–SBPF6 was determined according to AOAC Official Methods [47]. Moisture and ash contents were determined using Methods 925.10 and 923.03, respectively. Fat content was determined gravimetrically by Soxhlet extraction according to Method 920.39, with minor modifications. Briefly, approximately 3 g of each sample was extracted with hexane for 3 h, after which the solvent was removed by evaporation and the extracted lipids were quantified gravimetrically. Crude protein (CP) content was determined by the Kjeldahl method according to Method 978.04. Following digestion, distillation and titration, the nitrogen content was converted to crude protein using a nitrogen-to-protein conversion factor of 6.25. Total dietary fibre (TDF), insoluble dietary fibre (IDF), and soluble dietary fibre (SDF) were determined according to AOAC Method 991.43. Available carbohydrate content was calculated by difference as the percentage remaining after subtracting the measured contents of moisture, crude fat, crude protein, total dietary fibre and ash from 100 [40]. The energy value (kcal/100 g) was estimated using general Atwater conversion factors of 4 kcal/g for available carbohydrates and proteins, 9 kcal/g for fat and 2 kcal/g for dietary fibre [48].

### 2.7. Colour Measurements

The colour characteristics of SBP and its granulometric fractions (SBPF1–SBPF6) were determined in triplicate using a CR-5 Benchtop Colorimeter (Konica Minolta, Sakai, Osaka, Japan) operating in the CIELAB colour space. The colour coordinates $L^{*}$, $a^{*}$, and $b^{*}$ were recorded for each sample, where $L^{*}$ represents lightness, $a^{*}$ the red–green axis, and $b^{*}$ the yellow–blue axis. Positive and negative values of $a^{*}$ indicate redness and greenness, respectively, whereas positive and negative $b^{*}$ values correspond to yellowness and blueness, respectively [49]. Based on the recorded values, total colour intensity (TCI) was calculated according to Equation (9) [50]:

(9) $$TCI=\sqrt{{\left(L^{*}\right)}^{2}+{\left(a^{*}\right)}^{2}+{\left(b^{*}\right)}^{2}}$$

Colour saturation (chroma, C) and hue angle ($h^{{}^{\circ}}$), representing the dominant colour tone, were calculated using Equations (10) and (11), respectively [49]:

(10) $$C=\sqrt{{\left(a^{*}\right)}^{2}+{\left(b^{*}\right)}^{2}}$$

(11) $$h^{{}^{\circ}}=tan^{-1}\left(\frac{b^{*}}{a^{*}}\right)$$

The total colour difference (ΔE) of each granulometric fraction relative to the unfractionated reference sample (SBP) was calculated using the CIELAB colour space Equation (12), which accounts for simultaneous shifts in lightness, redness, and yellowness coordinates [49].

(12) $$\Delta E=\sqrt{{\left(L^{*}-L_{0}^{*}\right)}^{2}+{\left(a^{*}-a_{0}^{*}\right)}^{2}+{\left(b^{*}-b_{0}^{*}\right)}^{2}}$$

where $L^{*}$, $a^{*}$, and $b^{*}$ represent the colour coordinates of each granulometric fraction, whereas $L_{0}^{*}$, $a_{0}^{*}$, and $b_{0}^{*}$ correspond to those of the reference sample (SBP).

The ΔE values were interpreted according to the reported perceptibility scale [51]: ΔE < 1, imperceptible difference; 1 ≤ ΔE < 2, perceptible only to experienced observers; 2 ≤ ΔE < 3.5, noticeable difference; 3.5 ≤ ΔE < 5, clear difference; and ΔE ≥ 5, very clear difference.

### 2.8. Analytical Procedures for Total Phenolic Content, Total Flavonoid Content, DPPH Radical Scavenging Activity, and Ferric Reducing Antioxidant Power (FRAP)

#### 2.8.1. Preparation of Ethanolic Sample Extracts

Ethanolic extracts were prepared following the methodology reported in [52], using 70% (*v*/*v*) ethanol and two consecutive extraction steps. For each independent experimental replicate, extraction was performed in triplicate. Each sample was first homogenized using a mortar and pestle, after which three separate 1.0 g aliquots were individually mixed with 10 mL of 70% ethanol and extracted for 2 h at room temperature under continuous shaking (SK-L330-Pro linear shaker, Ningbo Scientz Biotechnology Co., Ltd., Ningbo, China). The mixtures were centrifuged at 10,000 rpm for 10 min (EBA 21 centrifuge, Hettich, Tuttlingen, Germany). The residual solids were re-extracted with fresh solvent for an additional 1 h under the same conditions, followed by centrifugation. The supernatants from both extraction cycles were combined prior to storage at −20 °C in the dark. The resulting ethanolic extracts were subsequently used to determine total phenolic content, total flavonoid content, DPPH radical-scavenging activity, and ferric reducing antioxidant power (FRAP).

#### 2.8.2. Total Phenolic Content Using the Folin–Ciocalteu Assay

The total phenolic content (TPC) of the ethanolic extracts obtained from SBP and SBPF1–SBPF6 was determined using the Folin–Ciocalteu colorimetric assay [53]. Prior to analysis, the extracts were diluted 1:25 (*v*/*v*) with 70% ethanol. For the assay, 0.5 mL of each diluted extract was mixed with 2.5 mL of Folin–Ciocalteu reagent previously diluted (1:10, *v*/*v*) with distilled water. After 5 min, 2.0 mL of 7.5% (*w*/*v*) sodium carbonate solution was added. The reaction mixtures were incubated for 30 min at 50 °C in a thermostatically controlled oven (AIR Concept, Precisa Gravimetrics AG, Dietikon, Switzerland), and the absorbance was measured at 750 nm using a T60 UV–Visible spectrophotometer (PG Instruments Ltd., Leicestershire, UK). A reagent blank was prepared by replacing the extract with 70% ethanol. TPC was calculated from a gallic acid calibration curve and expressed as milligrams of gallic acid equivalents per 100 g dry weight (mg GAE/100 g d.w.).

#### 2.8.3. Total Flavonoid Content Using the Aluminum Chloride Colorimetric Assay

The total flavonoid content (TFC) of the extracts was assessed using the colorimetric method previously outlined in [54]. Briefly, 3.0 mL of each extract was mixed with 4.5 mL of distilled water and 1.0 mL of 0.3% (*w*/*v*) sodium nitrite (NaNO_2_). After 6 min at 20 °C, 1.0 mL of 10% aluminium nitrate [Al(NO_3_)_3_] was added, followed by a further 6 min incubation under the same conditions. Subsequently, 10 mL of 4% (*w*/*w*) NaOH was added, and the final volume was adjusted to 25 mL with 70% ethanol. Following a 15 min reaction period, the sample absorbance was measured at 510 nm, with 70% ethanol used as the blank. A quercetin standard curve (0.5–50 µg/mL) was used for TFC quantification, and the results were reported as mg quercetin equivalents (QE)/100 g d.w.

#### 2.8.4. Free Radical Scavenging Activity Using the DPPH Assay

The free radical scavenging activity of the ethanolic extracts obtained from SBP and SBPF1–SBPF6 was assessed using the 2,2-diphenyl-1-picrylhydrazyl (DPPH) assay according to Difonzo et al. [55]. The method is based on the ability of antioxidant compounds present in the ethanolic extracts to reduce the stable DPPH radical, resulting in a decrease in absorbance proportional to the free radical scavenging activity. Prior to analysis, the extracts were diluted 1:5 (*v*/*v*) with 70% ethanol. Briefly, 1.0 mL of each diluted extract was mixed with 2.5 mL of 0.1 mM DPPH solution and vortexed thoroughly. The reaction mixtures were incubated in the dark at 20 °C for 30 min, after which the absorbance was measured at 517 nm. A control was prepared by replacing the extract with 70% ethanol, while 70% ethanol was used as the blank. The DPPH radical scavenging activity was calculated as percentage inhibition relative to the control. A Trolox calibration curve, obtained by plotting DPPH radical scavenging activity (%) against Trolox concentration within the range of 1.0–25 µg/mL, was used to express the scavenging activity of the samples as µmol Trolox equivalents (TE)/g d.w. [40].

#### 2.8.5. Ferric-Reducing Antioxidant Power (FRAP)

The ferric-reducing antioxidant power of the ethanolic extracts obtained from SBP and SBPF1–SBPF6 was evaluated by the FRAP assay following the previously reported procedure [56]. The assay is based on the reduction of the ferric ion–TPTZ complex to the ferrous form by antioxidant compounds present in the ethanolic extracts under acidic conditions, resulting in the formation of a blue Fe^2+^–TPTZ complex with maximum absorbance at 593 nm. The FRAP reagent was freshly prepared by mixing acetate buffer (pH 3.6), 10 mM TPTZ solution prepared in 40 mM HCl, and 20 mM ferric chloride hexahydrate solution at a ratio of 10:1:1 (*v*/*v*/*v*). Prior to analysis, the ethanolic extracts were diluted 1:50 (*v*/*v*) with 70% ethanol. A volume of 0.5 mL of each diluted extract was combined with 2.5 mL of freshly prepared FRAP reagent and incubated at 37 °C for 30 min in the dark. After incubation, the reaction mixtures were assessed by measuring absorbance at 593 nm, using 70% ethanol as the reference blank. FRAP values were calculated from a calibration curve prepared with ferrous sulphate heptahydrate and expressed as µmol Fe^2+^ equivalents per g d.w.

### 2.9. Structural Characterization of Seed-Free Sea Buckthorn Pomace and Its Granulometric Fractions by ATR-FTIR Spectroscopy

Attenuated total reflectance Fourier-transform infrared (ATR-FTIR) spectroscopy was employed to identify functional groups and evaluate structural features of SBP and its granulometric fractions (SBPF1–SBPF6). Infrared spectra were acquired at room temperature using a Nicolet™ iS50 FTIR spectrometer (Thermo Fisher Scientific, Waltham, MA, USA) equipped with a diamond ATR accessory. Before spectral analysis, the powdered samples were dried overnight at 40 °C to minimize the influence of residual moisture while preserving sample integrity. The dried powders were directly placed onto the ATR crystal, and spectra were collected over the wavenumber range of 4000–400 cm^−1^ using 32 scans at a spectral resolution of 4 cm^−1^ [57]. The recorded spectra were expressed as transmittance (%) as a function of wavenumber (cm^−1^) and comparatively evaluated to identify differences in the spectral features associated with functional groups between SBP and its granulometric fractions. The spectral data were analyzed using the “Find Peaks” function of the OMNIC software (version 9.16.188, Thermo Fisher Scientific, Waltham, MA, USA), with peak detection performed automatically over the 4000–400 cm^−1^ spectral region. The corresponding peak positions, intensities, and integrated areas were obtained from the generated peak lists.

### 2.10. Structural Investigation of Seed-Free Sea Buckthorn Pomace and Its Granulometric Fractions by Wide-Angle X-Ray Scattering (WAXS)

Wide-angle X-ray scattering (WAXS) was used to compare the scattering profiles of SBP and its granulometric fractions (SBPF1–SBPF6), with particular attention to the position and distribution of the detected scattering maxima, expressed in terms of scattering angle (2θ), as qualitative indicators of differences in structural ordering, as well as to the estimated crystallite size, following approaches previously applied for the structural characterization of plant-derived materials [57,58].

Measurements were performed at room temperature using a Xenocs Xeuss 3.0 system (Xenocs SAS, Grenoble, France) equipped with a Cu Genix 3D X-ray source and a Dectris Eiger2 Si 1M detector (Dectris, Baden-Dättwil, Switzerland). The samples were analyzed under vacuum conditions using Kapton film as a support material. The sample-to-detector distance was set at 45 mm, with H-Flux collimation and an acquisition time of 300 s [40,57]. The collected scattering data were processed using XSACT software (version 2.10; Xenocs, Grenoble, France) [59].

The resulting WAXS profiles were comparatively evaluated by examining the detected peak positions in SBP and SBPF1–SBPF6. In addition, the average crystallite size, expressed in Ångströms (Å), was calculated using the Scherrer Equation (13) [60], based on the broadening of three selected reflections located at approximately 15°, 20°, and 33° (2θ).

(13) $$D(Å)=\frac{Κ\times \lambda }{\beta \times cos\theta }$$

where D is the average crystallite size, K is the shape factor (0.9), λ is the wavelength of the X-ray radiation (1.54056 Å for Cu Kα1 radiation), β is the full width at half maximum of the selected diffraction peaks expressed in radians, and θ is the corresponding Bragg angle, corresponding to half of the experimentally recorded 2θ value.

Peak analysis was carried out using X’Pert HighScore Plus software (version 2.2b, PANalytical B.V., Almelo, The Netherlands) [61].

### 2.11. Statistical Analysis

Results are presented as mean ± standard deviation (SD) based on three independent experimental replicates (*n* = 3). For each independent experimental replicate, each granulometric fraction was analyzed in triplicate, and the mean of the three analytical determinations was used as the value for that experimental replicate. Differences among SBP and its granulometric fractions (SBPF1–SBPF6) were evaluated using one-way analysis of variance (ANOVA), followed by Tukey’s post hoc multiple comparison test. Prior to ANOVA, the data were checked for homogeneity of variances using Levene’s test and for normality of the data distribution. Statistical differences were considered significant at *p* < 0.05. The mean values of the compositional, colour, technofunctional, phytochemical, and antioxidant parameters obtained for SBP and SBPF1–SBPF6 were compiled into a common dataset and analysed using Pearson correlation, principal component analysis (PCA), and agglomerative hierarchical clustering (AHC) using PAST software (version 5.3) [62]. Pearson correlation coefficients (r) were calculated with statistical significance set at *p* < 0.05. PCA was based on the correlation matrix to account for differences in measurement units and scales [63], and PC1 and PC2 were retained for graphical representation. For AHC, variables were standardized by z-score transformation, and clustering was performed using Ward’s minimum-variance method with Euclidean distance; the resulting relationships among samples were visualized in a dendrogram [64].

## 3. Results and Discussion

### 3.1. Particle-Size Profile of the Samples

**Figure 2:**
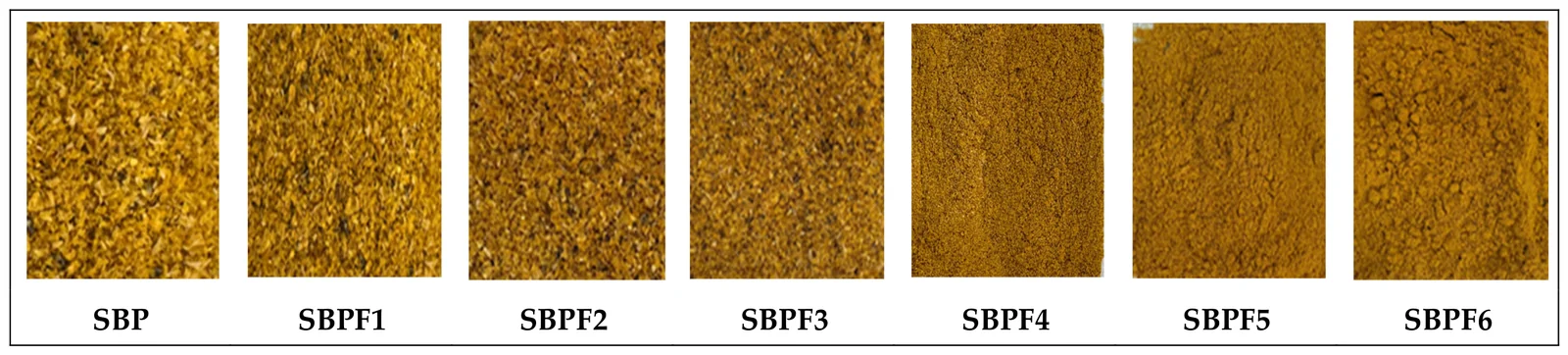
Seed-free sea buckthorn pomace and its granulometric fractions obtained by grinding and sieving. SBP: unfractionated sea buckthorn pomace; SBPF1–SBPF6: granulometric fractions with particle sizes of >2 mm, 1–2 mm, 0.5–1 mm, 0.25–0.5 mm, 0.15–0.25 mm, and 0–0.15 mm, respectively.

Figure 2 illustrates the seed-free sea buckthorn pomace (SBP) and the granulometric fractions (SBPF1–SBPF6) obtained by grinding followed by successive sieving.

The particle-size distribution and granulometric characteristics of the obtained fractions are presented in Table 1, which summarizes the sieve opening ($l_{i}$), average particle size ($d_{i}$), percentage of material with particle sizes between two adjacent sieve openings ($a_{i}$), corresponding to the proportion of material within each particle-size class, and the cumulative percentages of material with particle sizes smaller ($T_{i}$) and larger ($R_{i}$) than the corresponding sieve opening.

The grinding–sieving procedure separated the seed-free sea buckthorn pomace into six granulometric fractions with distinct particle-size ranges. The highest proportions of material were recovered in the 0.25–0.5 mm (24.27%) and 0.5–1 mm (26.09%) fractions, which together accounted for 50.36% of the fractionated material. The finest fraction (0–0.15 mm) represented 10.52%, whereas the coarsest fraction (>2 mm) accounted for 9.35%. Thus, the 0.25–1 mm particle-size interval represented the predominant size range obtained through dry fractionation by grinding–sieving and is expected to contribute substantially to the physicochemical and functional properties of the fractionated pomace.

The predominance of the intermediate granulometric fractions may be related to the heterogeneous structure of SBP, which contains tissue fragments with different mechanical properties and resistance to fragmentation. During grinding, these structural differences may result in unequal breakage of the plant matrix and consequently in the accumulation of material within specific particle-size classes. The relatively low proportion of the finest fraction indicates that the applied grinding conditions did not result in excessive production of ultrafine particles. The obtained particle-size profile therefore reflects both the applied grinding–sieving conditions and the heterogeneous nature of the SBP matrix. Similar observations have been reported for other plant-based materials subjected to granulometric fractionation. Successive sieving has been shown to effectively separate blackthorn berry powder into distinct fractions, with the resulting particle-size distribution influenced by sieve characteristics, vibration conditions, and the physical properties of the plant material [25]. Comparable findings indicate that dry fractionation by grinding–sieving of plant material generates fractions with distinct particle-size distributions, although separation efficiency may be affected by particle adhesion, agglomeration, and differences in material structure [11]. These observations support the present results, indicating that particle-size distribution is influenced by the applied grinding–sieving conditions and the intrinsic heterogeneity of the plant matrix. This procedure effectively separated seed-free sea buckthorn pomace into six distinct granulometric fractions, enabling subsequent evaluation of their compositional, phytochemical, structural, and technofunctional properties.

### 3.2. Proximate Composition of Seed-Free Sea Buckthorn Pomace and Its Granulometric Fractions

The proximate composition of the seed-free sea buckthorn pomace (SBP) and its granulometric fractions (SBPF1–SBPF6) is presented in Table 2.

Significant differences (*p* < 0.05) were observed among SBP and its granulometric fractions for all analyzed components, indicating compositional differentiation following granulometric fractionation. Residual moisture content remained low in all samples, ranging from 4.12% in SBPF6 to 5.49% in SBPF4. The relatively narrow moisture range indicates that the compositional differences observed among the samples were not primarily driven by differences in water content.

Crude protein (CP) content varied among the granulometric fractions, ranging from 5.59 to 6.48%. The highest values were recorded in SBPF1 and SBPF2, which did not differ significantly from each other (*p* > 0.05), while SBPF3 showed an intermediate value. SBP and SBPF4 also showed comparable CP contents, whereas significantly lower values were observed in SBPF5 and SBPF6. These results indicate that protein was distributed differently among the particle-size classes, with no enrichment in the smallest fractions. Similar variations in protein distribution among granulometric fractions have been reported for fruit-derived powders, where differences among fractions were related to tissue fragmentation behaviour and matrix structure during grinding [25]. The distribution of proteins after dry fractionation may therefore depend on the specific characteristics of the plant material, as also reported for other plant matrices [11].

Fat content followed a different distribution pattern from crude protein across the granulometric fractions, with significant differences among all samples (*p* < 0.05). The lowest value was recorded in SBPF1 (6.82%), followed by a progressive increase with decreasing particle size, reaching 9.35% in SBPF6, whereas the unfractionated SBP contained 8.25%. This pattern indicates that lipid components were unevenly distributed among the particle-size classes, with higher fat contents in the finer fractions. Similar variations in lipid distribution among granulometric fractions have been reported for plant-derived powders and have been related to the structural characteristics of the raw material and its fragmentation behaviour during size reduction [11,25]. The fat contents obtained in the present study were within the ranges previously reported for sea buckthorn pomace [65,66]. Differences among studies may be related to processing conditions and the presence or absence of residual seed material.

Total dietary fibre (TDF) was the predominant component in all samples, ranging from 42.19 to 58.46%, with significant differences among all samples (*p* < 0.05). The highest content was observed in SBPF1, followed by a gradual decrease with decreasing particle size, reaching the lowest value in SBPF6, whereas the unfractionated sample contained 49.61%. These results indicate that dietary fibre was unevenly distributed among the granulometric fractions, with the highest content recorded in the coarsest fraction, in agreement with previous observations for fruit- and plant-derived powders [11,25]. Previous studies reported TDF contents of 63.61 and 55.80 g/100 g d.w. for sea buckthorn pomace [65,67]. Since insoluble dietary fibre represents the predominant fibre fraction in sea buckthorn pomace [66,67], the higher TDF content observed in SBPF1 may reflect a greater contribution of structural cell-wall material in this fraction. The distribution of dietary fibre differed among the granulometric fractions, with insoluble dietary fibre (IDF) decreasing progressively from the coarsest to the finest fractions, whereas soluble dietary fibre (SDF) exhibited the opposite trend, increasing towards the finer fractions. Consequently, the IDF/SDF ratio declined with decreasing particle size, while SBP displayed an intermediate profile. These findings indicate that granulometric separation not only redistributed total dietary fibre among the fractions but also resulted in different relative proportions of its insoluble and soluble components. A similar trend was reported for olive pomace, where finer granulometric fractions showed lower IDF and higher SDF contents, while further particle-size reduction accentuated this shift towards the soluble fibre fraction [28]. The predominance of the IDF fraction observed in the present study is consistent with previous reports for sea buckthorn pomace, in which IDF was also the major dietary fibre fraction [66,68]. Although the IDF/SDF ratio differed among studies, reflecting differences in raw material and processing conditions, IDF remained the predominant fibre fraction across the reported samples.

Ash content ranged from 2.33 to 2.78%, with significant differences observed among most samples (*p* < 0.05). SBP and SBPF4 did not differ significantly from each other, whereas the remaining fractions showed significant differences. A previous study reported an ash content of 1.38 g/100 g d.w. for sea buckthorn pomace powder [66]. Such differences may reflect variations in the intrinsic mineral composition of the raw material and in pomace composition, particularly the presence or absence of seeds.

Available carbohydrates (CRB) content differed significantly among all samples (*p* < 0.05), increasing progressively from 21.27% in SBPF1 to 35.97% in SBPF6, while the unfractionated SBP contained 28.52%. This trend was opposite to that observed for total dietary fibre, which decreased with decreasing particle size. The energy value also differed significantly among all samples, increasing from 289.30 kcal/100 g in SBPF1 to 334.77 kcal/100 g in SBPF6, while SBP showed an intermediate value. This variation followed the changes observed for fat, CRB, and TDF content, with the finest fraction showing the highest energy value and the coarsest fraction the lowest.

Granulometric fractionation resulted in a differentiated distribution of the main compositional components of SBP. The coarser granulometric fractions, particularly SBPF1, exhibited higher TDF content and lower fat, CRB, and energy values, whereas the finest fraction (SBPF6) showed the lowest TDF and CP contents, together with the highest fat, CRB, and energy values. These differences demonstrate that dry fractionation can generate fractions with distinct compositional profiles, providing a basis for selecting sea buckthorn pomace fractions according to targeted nutritional characteristics.

### 3.3. Technofunctional Properties of Seed-Free Sea Buckthorn Pomace and Its Granulometric Fractions

The technofunctional properties presented in Table 3 include water swelling capacity (WSC), water retention capacity (WRC), water solubility index (WSI), and oil retention capacity (ORC) of SBP and its granulometric fractions (SBPF1–SBPF6).

These parameters were evaluated to assess how granulometric fractionation affected the hydration and oil-retention behaviour of the resulting fractions compared with the unfractionated SBP.

WSC did not change progressively with decreasing particle size, ranging from 1.98 to 3.51 mL/g across the fractions. The highest value was recorded in SBPF3, which was significantly higher than that of SBP and all other fractions (*p* < 0.05). WSC decreased in the finer fractions, with no significant difference between SBPF5 and SBPF6. Similar observations have been reported for structurally modified sea buckthorn pomace, where swelling behaviour was related to changes in cell-wall polysaccharides and fibre organization [46]. The observed variation in WSC suggests that swelling capacity is influenced by both particle size and the structural characteristics of the matrix.

Water retention capacity (WRC) differed significantly among all samples (*p* < 0.05), ranging from 1.28 g/g in SBPF1 to 2.68 g/g in SBPF5. WRC increased progressively with decreasing particle size from SBPF1 to SBPF5, followed by a significant decrease in SBPF6. The lower WRC of the coarser fraction may be associated with its lower specific surface area and reduced accessibility of hydrophilic groups, whereas particle-size reduction can increase the exposed surface and facilitate interactions between water and hydroxyl groups of cell-wall polysaccharides. Jurevičiūtė et al. [66] reported higher hydration properties for whole berry pomace powders, including sea buckthorn pomace, which may be related to differences in material composition, particularly the presence of seeds. Their samples also contained high levels of TDF (49.24–72.67 g/100 g d.w.), including 63.61 g/100 g d.w. for sea buckthorn pomace, which may also contribute to the hydration behaviour of the material. The fibre distribution observed among the granulometric fractions may also have contributed to this behaviour. As particle size decreased, the proportion of SDF increased, whereas that of IDF declined, resulting in a progressive decrease in the IDF/SDF ratio. Soluble dietary fibre is characterized by a high water-holding capacity and contributes to moisture retention, whereas insoluble fibre contributes to water absorption through the insoluble fibre matrix [40]. Richards et al. [69] also showed that changes in the insoluble-to-soluble fibre balance can influence hydration-related properties of plant pomaces. Accordingly, the decreasing IDF/SDF ratio may have contributed to the increasing trend in WRC from SBPF1 to SBPF5.

The water solubility index (WSI) differed significantly among all samples (*p* < 0.05) and increased progressively with decreasing particle size, ranging from 7.03% in SBPF1 to 12.59% in SBPF6. The lower WSI of the coarser fractions may be related to their smaller specific surface area and more limited disruption of plant tissues, which can restrict the release of water-soluble constituents. With decreasing particle size, the greater exposed surface and increased cell-wall disruption may facilitate the release of soluble compounds into the aqueous phase, explaining the progressive increase in WSI. This trend indicates that particle-size reduction favoured the solubilization of constituents from the SBP matrix. However, WSI is also influenced by the composition and structural organization of the fibre matrix. Previous findings showed that structural modification of sea buckthorn pomace affected water solubility through changes in cell-wall polysaccharides and fibre organization [46], supporting the combined influence of particle size and matrix characteristics on this property. The progressive decrease in the IDF/SDF ratio towards the finer fractions may be associated with the corresponding increase in WSI. Speroni et al. [28] similarly reported greater solubility in finer olive pomace fractions following particle-size reduction, while SDF fractions isolated from berry pomaces exhibited higher WSI than the corresponding IDF fractions [68].

Oil retention capacity (ORC) showed an opposite trend to WRC and WSI, decreasing with decreasing particle size. The highest values were recorded for SBPF1 and SBPF2 (2.96 and 2.91 g/g, respectively), with no significant difference observed (*p* > 0.05), whereas ORC decreased significantly towards the finer fractions. The lowest values were observed in SBPF5 and SBPF6, which also did not differ significantly from each other. This pattern suggests that oil retention was favoured in the coarser fractions and may reflect differences in fibre composition and structural organization among the particle-size classes. Kaminskytė et al. [46] reported an ORC of 1.63 g/g for untreated sea buckthorn pomace and 2.16–3.08 g/g after enzymatic modification, while Jurevičiūtė et al. [66] reported an oil-binding capacity of 1.09 g/g for sea buckthorn pomace powder. The IDF/SDF distribution is also consistent with this pattern. The coarser fractions contained the highest relative proportions of IDF and exhibited the highest ORC, whereas both IDF contribution and ORC decreased towards the finer fractions. Jagelavičiūtė et al. [68] reported that the oil-retention behaviour of SDF and IDF varied among the berry materials investigated; for sea buckthorn pomace, IDF exhibited a higher ORC than SDF. This agreement suggests that the changing balance between insoluble and soluble fibre may have contributed to the differences in oil retention among the granulometric fractions.

Granulometric fractionation produced SBP fractions with distinct technofunctional characteristics. WSC was highest in SBPF3, WRC in SBPF5, WSI increased progressively towards the finest fraction, whereas ORC was favoured in the coarser fractions. These different technofunctional responses may reflect both particle-size differences and the changing relative contributions of IDF and SDF among the granulometric fractions. Previous studies have also shown that changes in fibre organization can influence the hydration-related properties of sea buckthorn pomace [46]. This functional differentiation is consistent with previous evidence [19] and provides a basis for relating the individual SBP fractions to broader particle-size ranges and potential food applications.

SBPF6 (<150 µm), which exhibited the highest WSI, corresponds to particle-size ranges investigated in beverage and semi-liquid food systems. He et al. [30] reported improved dispersion-related characteristics for water dropwort powders below 150 µm, while carrot pomace powders of 57–138 µm were incorporated into functional milk drinks, where particle size affected water- and oil-holding properties, viscosity, turbidity, and sedimentation behaviour [31]. Particle size also influenced grittiness and particle perception of apple pomace powders in yogurt-type systems [32]. The high WSI and fine particle size of SBPF6 therefore suggest potential for further evaluation in beverages and semi-liquid foods. Previous studies also indicate that particle-size reduction does not uniformly improve hydration-related properties, which are also influenced by matrix composition, structure, and processing conditions [31,70].

SBPF4 and SBPF5 (150–500 µm), which showed relatively high WRC, correspond to particle-size ranges investigated in bakery and cereal-based applications. Jose et al. [33] evaluated pineapple pomace fractions of 400–251 and 250–150 µm, while Rocha Parra et al. [36] investigated apple pomace particles of approximately 362–482 µm, reporting effects of particle size on hydration, dough behaviour, and texture. Similar effects were reported for pitaya peel powders with median particle sizes of approximately 416 and 184 µm [34]. Troilo et al. [35] showed that grape pomace fractions of approximately 150–600 µm incorporated into muffins affected phenolic content, antioxidant activity, texture, crumb structure, and sensory properties, while particle-size-fractionated banana peel powder influenced the physicochemical and sensory characteristics of instant noodles [71]. These findings support further evaluation of SBPF4 and SBPF5 in bakery and cereal-based matrices. The coarser fractions SBPF1–SBPF3 (>500 µm), which exhibited higher ORC, may be suitable for further evaluation in structured, fibre-rich food systems where larger particles can be incorporated. This potential application is supported by the effects of coarse apple pomace particles on water retention, dough rheology, texture, and sensory characteristics reported by Rocha Parra et al. [36].

### 3.4. Colour Properties of Seed-Free Sea Buckthorn Pomace and Its Granulometric Fractions

The colour parameters of seed-free sea buckthorn pomace (SBP) and its granulometric fractions are presented in Table 4.

SBP: unfractionated seed-free sea buckthorn pomace; SBPF1–SBPF6: granulometric fractions with particle sizes of >2 mm, 1–2 mm, 0.5–1 mm, 0.25–0.5 mm, 0.15–0.25 mm, and 0–0.15 mm, respectively. $L^{*}$: lightness; $a^{*}$: red–green coordinate; $b^{*}$: yellow–blue coordinate; ΔE: total colour difference compared with the seed-free sea buckthorn pomace (control); C: chroma; $h^{{}^{\circ}}$: hue angle; TCI: total colour intensity. Values are expressed as mean ± standard deviation (SD) of three independent experimental replicates (*n* = 3). Different superscript letters (a–g) within the same row indicate statisticant differences among samples (*p* < 0.05, one-way ANOVA).

Lightness ($L^{*}$) was significantly influenced by granulometric fractionation and increased progressively from the coarsest to the finest fraction. The lowest $L^{*}$ value was recorded for SBPF1 (51.13), whereas SBPF6 showed the highest value (53.15). The intermediate fractions followed the same gradual trend with decreasing particle size (51.49–52.73), while SBP exhibited an intermediate value of 52.41. Thus, the finer fractions were slightly lighter than the coarser ones. A comparable $L^{*}$ value of 52.80 was reported for hot-air-dried sea buckthorn pomace [49], close to those obtained for SBP and the finer fractions in the present study. Zhang et al. [72] also reported that particle-size reduction influenced light reflection and, consequently, the lightness of blueberry pomace powders, which may explain the increase in $L^{*}$ observed towards the finer fractions in our study.

The redness coordinate ($a^{*}$) also increased progressively across the granulometric fractions, from 16.81 in SBPF1 to 18.27 in SBPF6, while SBP showed an intermediate value of 17.63. The positive $a^{*}$ values observed for all samples indicate a red contribution to the characteristic orange-red colour of sea buckthorn pomace. Zhou et al. [49] reported a decrease in $a^{*}$ from 30.49 in fresh sea buckthorn pomace to 24.52 after hot-air drying and associated this change with pigment-related modifications occurring during drying. Within the investigated fractions, however, the gradual increase in $a^{*}$ towards SBPF6 indicated that the red component became more pronounced as particle size decreased.

A similar particle-size-related trend was observed for the yellowness coordinate ($b^{*}$), which increased significantly from 43.62 in SBPF1 to 45.51 in SBPF6. All $b^{*}$ values were positive, indicating a strong yellow contribution to the colour of the samples. Although the increase was consistent across the granulometric fractions, the relatively narrow range of $b^{*}$ values indicates that the differences in yellowness were moderate.

The same pattern was reflected in the derived colour parameters. TCI increased from 69.28 in SBPF1 to 72.32 in SBPF6, while SBP showed an intermediate value of 71.33. Chroma (C) similarly increased from 46.75 to 49.04, indicating slightly greater colour saturation in the finer fractions. In contrast, the hue angle ($h^{{}^{\circ}}$) decreased gradually from 68.92° in SBPF1 to 68.13° in SBPF6, with SBP at 68.63°. Despite this statistically significant variation, the narrow range of $h^{{}^{\circ}}$ values indicates that all samples maintained a similar yellow–orange hue. Particle-size-dependent changes in colour have also been reported for muffins formulated with different grape pomace fractions, together with differences in phenolic content and antioxidant activity [35]. Similarly, successive sieving of blackthorn berry powder resulted in granulometric fractions differing in the UV–Vis absorbance of coloured compounds and in the distribution of bioactive constituents [25].

The total colour difference (ΔE), calculated relative to SBP, ranged from 0.47 for SBPF4 to 2.09 for SBPF1. According to the classification criteria reported in the literature [52], the differences observed for SBPF4 and SBPF5 (ΔE < 1) would not be perceptible to the human eye. Values between 1 and 2 were obtained for SBPF2, SBPF3, and SBPF6 and would generally be distinguishable only by experienced observers. SBPF1 showed the greatest colour difference (2.09), falling within the range in which the difference may also be perceived by an inexperienced observer. None of the fractions reached a ΔE value associated with a clearly perceptible colour difference (>3.5). SBPF4 and SBPF5 showed the greatest colour similarity to SBP, whereas SBPF1 exhibited the largest colour difference.

The colour results show a systematic variation across the granulometric fractions. Decreasing particle size was associated with higher $L^{*}$, $a^{*}$, $b^{*}$, C, and TCI values and a slight decrease in h°, resulting in finer fractions that were somewhat lighter and more saturated while retaining the characteristic yellow–orange hue of the material. The relatively low ΔE values further indicate that these differences remained visually limited, while the characteristic appearance of the original SBP was largely maintained after granulometric fractionation.

### 3.5. Phytochemical Content and Antioxidant Activity of SBP and Its Granulometric Fractions

#### 3.5.1. Total Phenolic Content

**Figure 3:**
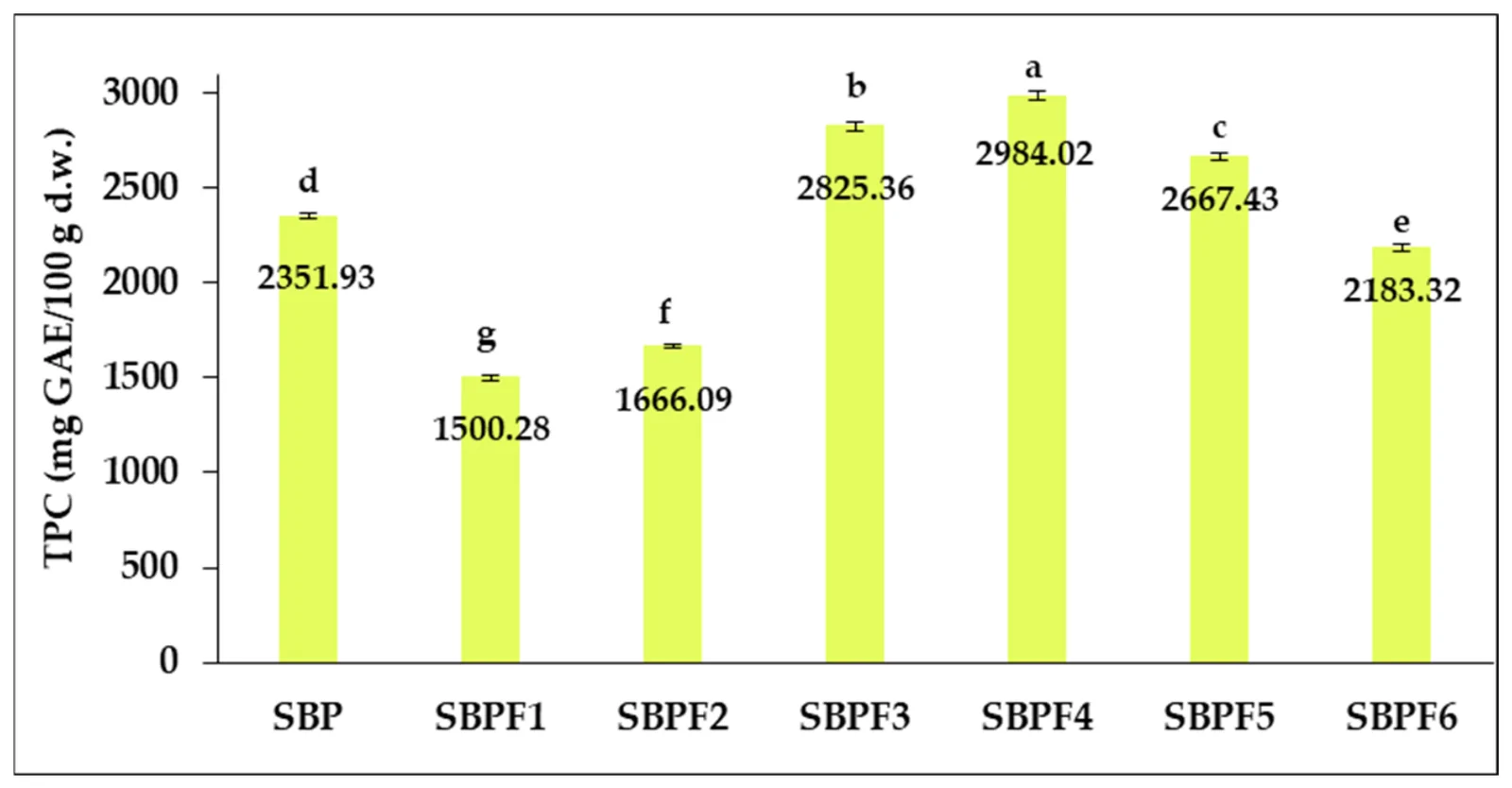
Total phenolic content (TPC) of seed-free sea buckthorn pomace and its granulometric fractions. SBP: seed-free sea buckthorn pomace; SBPF1–SBPF6: granulometric fractions with particle sizes > 2 mm, 1–2 mm, 0.5–1 mm, 0.25–0.5 mm, 0.15–0.25 mm, and 0–0.15 mm, respectively. Values are expressed as mean ± standard deviation (SD) of three independent experimental replicates (*n* = 3). Bars labelled with different letters (a–g) indicate significant differences among samples (*p* < 0.05, one-way ANOVA).

Figure 3 presents the total phenolic content (TPC) of seed-free sea buckthorn pomace (SBP) and its granulometric fractions obtained by grinding followed by successive sieving.

TPC varied significantly among SBP and the six granulometric fractions (*p* < 0.05), with values ranging from 1500.28 to 2984.02 mg GAE/100 g d.w. The unfractionated SBP contained 2351.93 mg GAE/100 g d.w. The most pronounced enrichment occurred in SBPF4, which reached 2984.02 mg GAE/100 g d.w., followed by SBPF3 (2825.36 mg GAE/100 g d.w.) and SBPF5 (2667.43 mg GAE/100 g d.w.). These values were 26.88%, 20.13%, and 13.41% higher than those of SBP, respectively. By comparison, SBPF1 and SBPF2 showed substantially lower TPC values of 1500.28 and 1666.09 mg GAE/100 g d.w., corresponding to decreases of 36.21% and 29.16%. Although SBPF6 represented the finest fraction, its TPC was 2183.32 mg GAE/100 g d.w., 7.17% lower than that of SBP. Therefore, the highest phenolic contents were associated with the intermediate granulometric fractions rather than with the finest particles.

This behaviour is consistent with observations reported for other fractionated plant materials. In blackthorn berry powder, the highest TPC occurred in fractions between 0.315 and 0.630 mm, whereas the coarsest fraction contained approximately 1.6-fold less phenolic material [25]. Similarly, TPC and antioxidant capacity did not follow a consistent particle-size trend in flamboyant-mirim fractions [19], while intermediate particle-size classes have also been reported to contain higher phenolic levels than the finest fractions [11]. These comparisons indicate that reducing particle size alone does not necessarily increase the amount of extractable phenolic compounds recovered from a plant powder.

The lower TPC observed in SBPF6 may be associated with the greater susceptibility of very fine particles to processing-related thermal and oxidative effects. Literature evidence from other plant materials indicates that intensive grinding can generate localized heating, which may contribute to the degradation of heat-sensitive phenolic compounds [73]. Similar particle-size-dependent reductions in TPC during grinding have also been reported for medicinal plant powders [12,18].

The TPC obtained for SBP was close to the value of 2265.99 mg GAE/100 g d.w. previously reported for sea buckthorn pomace [74]. SBPF3–SBPF5 exceeded this literature value, whereas SBPF1, SBPF2, and SBPF6 were lower. A higher value of 3255 mg GAE/100 g d.w. has also been reported [75], while TPC values of 2418–3019 mg GAE/100 g d.w. were found in other sea buckthorn pomace powders [76], corresponding more closely to the intermediate fractions obtained in the present study. Differences in TPC among peel-, pomace-, and seed-based sea buckthorn powders have also been described [77], showing that the composition of the starting material can strongly influence the phenolic profile. Values of 816.58 and 1394.98 mg GAE/100 g d.w. have also been reported [78], both lower than those measured for SBP and all granulometric fractions in the present study. Such variation among studies may reflect differences in cultivar, raw material composition, processing conditions, extraction procedures, and analytical methods.

Granulometric fractionation therefore altered the distribution of extractable phenolic compounds within the seed-free sea buckthorn pomace, with the strongest enrichment occurring in the intermediate fractions. The particularly high TPC of SBPF4 suggests that this fraction may be of interest when a phenolic-rich ingredient is desired for further valorisation.

#### 3.5.2. Total Flavonoid Content

**Figure 4:**
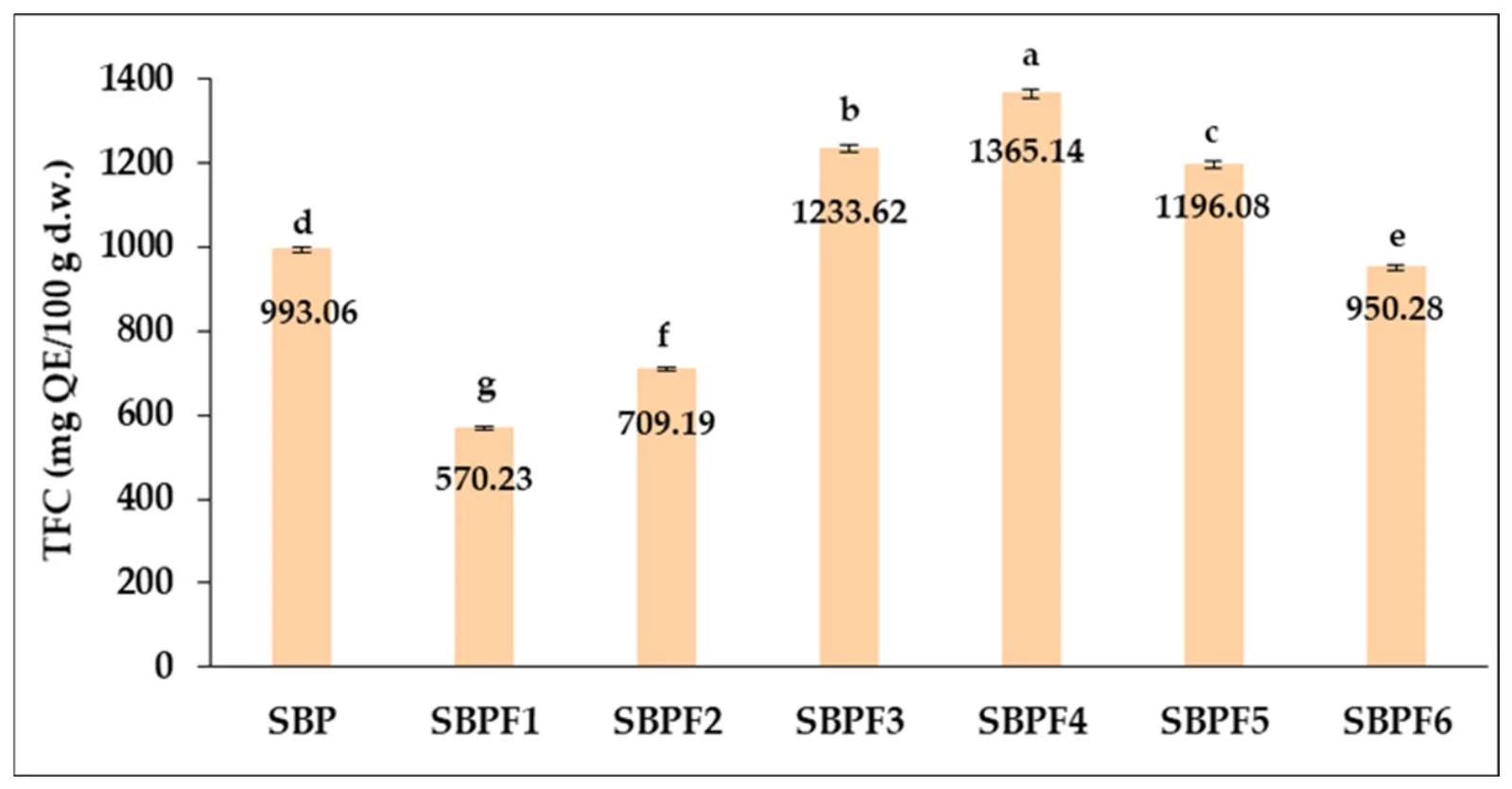
Total flavonoid content (TFC) of seed-free sea buckthorn pomace and its granulometric fractions. SBP: seed-free sea buckthorn pomace; SBPF1–SBPF6: granulometric fractions with particle sizes >2 mm, 1–2 mm, 0.5–1 mm, 0.25–0.5 mm, 0.15–0.25 mm, and 0–0.15 mm, respectively. Values are expressed as mean ± standard deviation (SD) of three independent experimental replicates (*n* = 3). Bars labelled with different letters (a–g) indicate significant differences among samples (*p* < 0.05, one-way ANOVA).

The TFC values obtained for SBP and its granulometric fractions are presented in Figure 4.

Significant differences (*p* < 0.05) were observed among all samples, with TFC ranging from 570.23 to 1365.14 mg QE/100 g d.w., compared with 993.06 mg QE/100 g d.w. in the unfractionated SBP. The highest TFC was recorded for SBPF4 (1365.14 mg QE/100 g d.w.), followed by SBPF3 (1233.62 mg QE/100 g d.w.) and SBPF5 (1196.08 mg QE/100 g d.w.), corresponding to increases of 37.47%, 24.22%, and 20.44%, respectively, relative to SBP. In contrast, SBPF1 and SBPF2 contained 570.23 and 709.19 mg QE/100 g d.w., representing decreases of 42.58% and 28.59%, respectively. The finest fraction, SBPF6, contained 950.28 mg QE/100 g d.w., 4.31% below the value of SBP. Thus, as observed for TPC, flavonoid content did not increase progressively with decreasing particle size, and the greatest enrichment occurred in the intermediate fractions.

A similar distribution pattern has been reported for particle-size-fractionated plant powders [11]. In *Hibiscus sabdariffa*, both TPC and TFC reached their highest values in an intermediate granulometric fraction, whereas the finest fraction did not show the greatest enrichment [11]. The comparable pattern observed in the present study, with SBPF3–SBPF5 showing the highest TFC values, indicates that granulometric separation produced fractions with different levels of extractable flavonoids. The TFC pattern also closely paralleled that observed for TPC, with the same intermediate fractions showing the highest values in both determinations.

For comparison, TFC values ranging from 6.57 to 9.01 mg QE/g f.w. have been reported for berries of four Romanian sea buckthorn cultivars [79], showing variation in flavonoid content among cultivars. Drying conditions may also influence the flavonoid composition of sea buckthorn material. Zhou et al. [49] reported changes in TFC following different drying treatments of sea buckthorn pomace and suggested that inhibition of enzymatic activity during drying may contribute to flavonoid retention. Differences in phytochemical composition have also been reported among sea buckthorn powders obtained from peel, marc, and seeds [77], indicating that the composition of the starting material can influence its bioactive profile.

The results therefore show that granulometric fractionation produced fractions with markedly different TFC. Flavonoid content increased from the coarser to the intermediate fractions, reaching a maximum in SBPF4, and subsequently decreased towards the finest fractions. The intermediate fractions, particularly SBPF4, showed the greatest flavonoid enrichment, indicating their potential for further valorisation as phenolic- and flavonoid-rich fractions of seed-free sea buckthorn pomace.

#### 3.5.3. DPPH Radical-Scavenging Activity and Ferric Reducing Antioxidant Power (FRAP)

**Figure 5:**
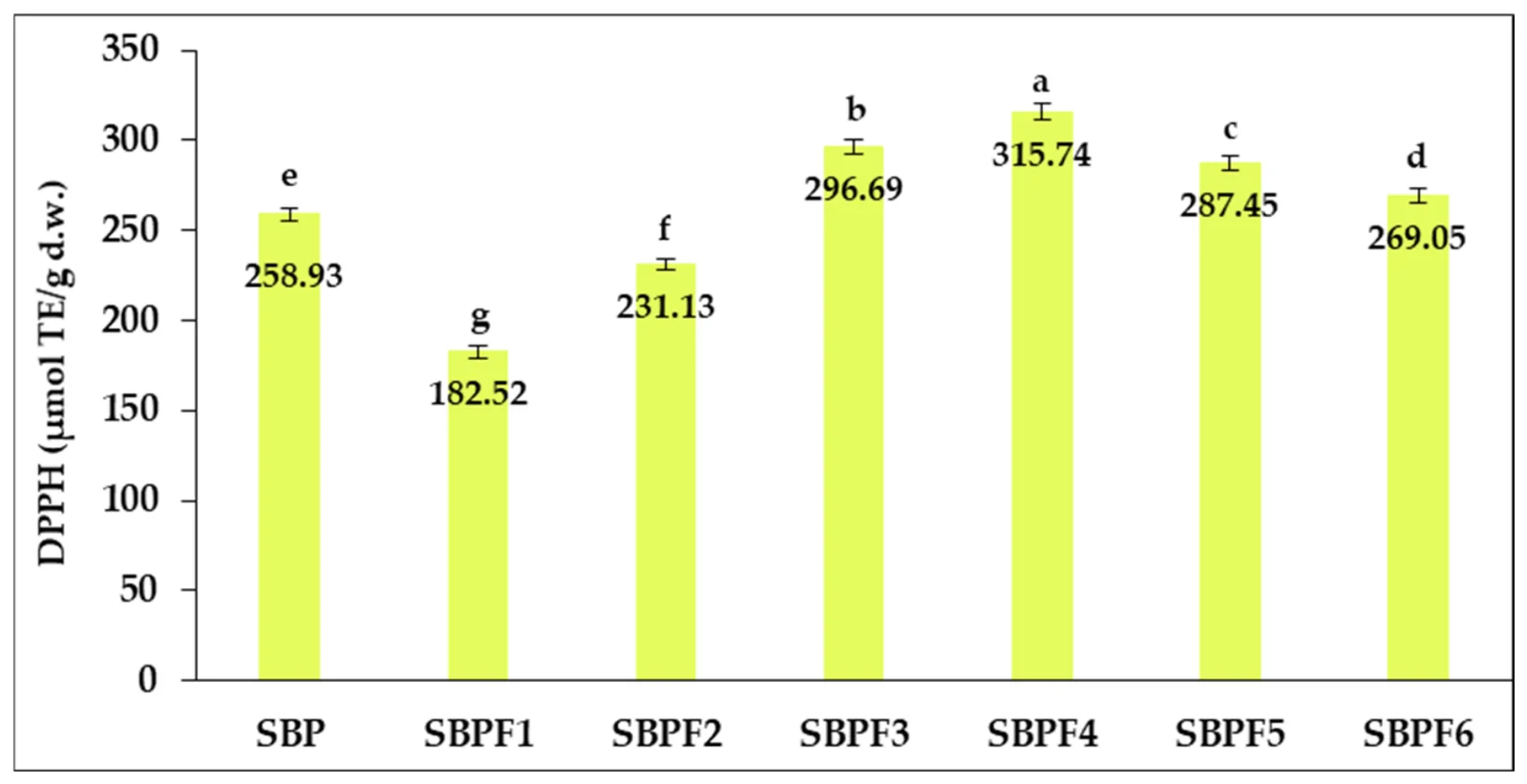
DPPH radical-scavenging activity of seed-free sea buckthorn pomace and its granulometric fractions. SBP: unfractionated sea buckthorn pomace; SBPF1–SBPF6: granulometric fractions with particle sizes >2 mm, 1–2 mm, 0.5–1 mm, 0.25–0.5 mm, 0.15–0.25 mm, and 0–0.15 mm, respectively. Values are expressed as mean ± standard deviation (SD) of three independent experimental replicates (*n* = 3). Bars labelled with different letters (a–g) indicate significant differences among samples (*p* < 0.05, one-way ANOVA).

The DPPH radical-scavenging activity of SBP and its granulometric fractions is presented in Figure 5.

Significant differences (*p* < 0.05) were observed among all samples, with values ranging from 182.52 to 315.74 μmol TE/g d.w., compared with 258.93 μmol TE/g d.w. for the unfractionated SBP.

The highest activity was recorded for SBPF4 (315.74 μmol TE/g d.w.), followed by SBPF3 (296.69 μmol TE/g d.w.) and SBPF5 (287.45 μmol TE/g d.w.), representing increases of 21.94%, 14.58%, and 11.01%, respectively, relative to SBP. SBPF1 and SBPF2 showed lower radical-scavenging activities of 182.52 and 231.13 μmol TE/g d.w., corresponding to decreases of 29.51% and 10.74%, respectively. The finest fraction, SBPF6, reached 269.05 μmol TE/g d.w., 3.91% above SBP, but remained below SBPF3–SBPF5. Thus, the strongest DPPH radical-scavenging activity was associated with the intermediate granulometric fractions rather than showing a continuous increase towards the finest fraction.

Previous studies have reported DPPH radical-scavenging activities of 55.66 μmol TE/g and 102.3–205.0 μmol TE/g for sea buckthorn pomace [10,80]. The unfractionated SBP in the present study exhibited higher DPPH activity than these reported values, while even greater radical-scavenging activity was observed in the intermediate fractions SBPF3–SBPF5.

**Figure 6:**
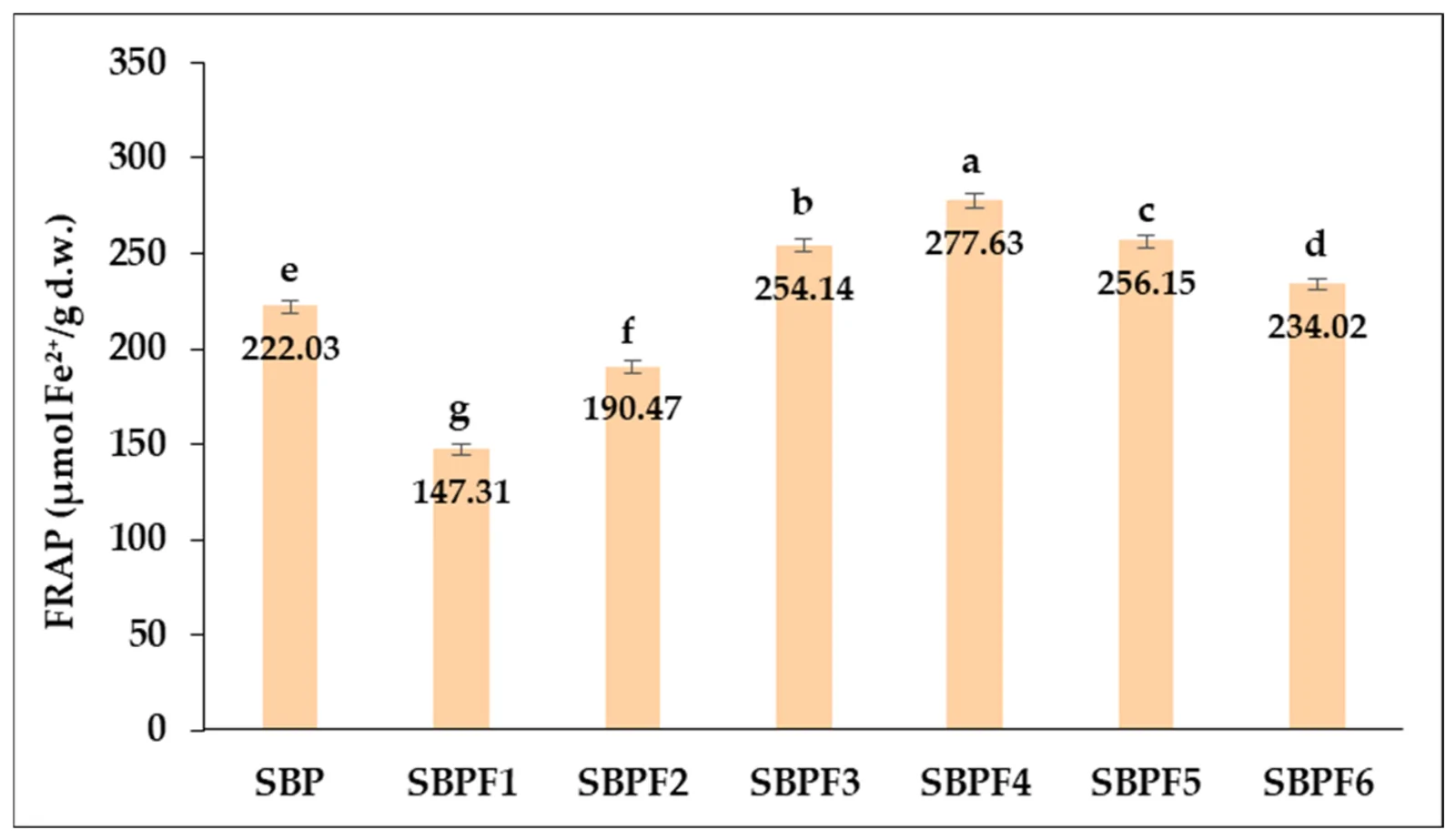
Ferric-reducing antioxidant power of seed-free sea buckthorn pomace and its granulometric fractions. SBP: unfractionated sea buckthorn pomace; SBPF1–SBPF6: granulometric fractions with particle sizes >2 mm, 1–2 mm, 0.5–1 mm, 0.25–0.5 mm, 0.15–0.25 mm, and 0–0.15 mm, respectively. Values are expressed as mean ± standard deviation (SD) of three independent experimental replicates (*n* = 3). Bars labelled with different letters (a–g) indicate significant differences among samples (*p* < 0.05, one-way ANOVA).

The FRAP assay showed the same general tendency, with the intermediate fractions exhibiting the greatest ferric reducing capacity (Figure 6).

FRAP values ranged from 147.31 to 277.63 μmol Fe^2+^/g d.w., compared with 222.03 μmol Fe^2+^/g d.w. for SBP. SBPF4 again showed the highest value (277.63 μmol Fe^2+^/g d.w.), followed by SBPF5 (256.15 μmol Fe^2+^/g d.w.) and SBPF3 (254.14 μmol Fe^2+^/g d.w.), corresponding to increases of 25.04%, 15.37%, and 14.46%, respectively. SBPF6 also exceeded SBP, reaching 234.02 μmol Fe^2+^/g d.w. (+5.40%). By contrast, SBPF1 and SBPF2 showed the lowest reducing capacities, with values of 147.31 and 190.47 μmol Fe^2+^/g d.w., representing decreases of 33.65% and 14.21%, respectively. As observed for DPPH, the greatest ferric reducing capacity was therefore found in the intermediate fractions.

Wu et al. [9] reported a substantially higher FRAP value of approximately 637.3 μmol Fe^2+^/g d.w. for air-dried whole sea buckthorn pomace than that obtained for the seed-free SBP in the present study (222.03 μmol Fe^2+^/g d.w.). This higher value may be related to raw material composition, including the presence of seeds in the material analysed, as well as to drying conditions and the extraction procedure [9].

DPPH and FRAP followed a similar pattern to TPC and TFC, with the intermediate fractions SBPF3–SBPF5 consistently showing the highest values and SBPF4 exhibiting the greatest activity. This similarity supports the contribution of phenolic constituents to the antioxidant properties of the fractions. Phenolic compounds, including flavonoids, are well known to contribute to antioxidant activity through hydrogen-atom and electron-donating mechanisms [81]. Isorhamnetin, quercetin, kaempferol, and their derivatives have also been identified among the major flavonoid constituents associated with the antioxidant capacity of sea buckthorn pomace [82].

Comparable effects of granulometric fractionation on antioxidant activity have been reported for other plant materials [11,25]. Significant differences in DPPH and ABTS radical-scavenging activities were found among granulometric fractions of *Boscia senegalensis*, *Dichrostachys glomerata*, and *Hibiscus sabdariffa*, although the fractions with the greatest antioxidant activity differed among species [11]. In blackthorn berry powder, intermediate granulometric fractions also showed higher DPPH radical-scavenging activity than the coarsest fraction [25]. These studies indicate that the effect of granulometric separation on antioxidant activity depends on the composition of the plant material and is not determined by particle size alone.

Grinding can increase the accessibility of intracellular compounds through disruption of plant tissues, while subsequent sieving separates particles according to their physical characteristics [81,83]. Similar effects of grinding and successive sieving on the distribution and accessibility of bioactive compounds have been discussed for other plant-derived powders [11,12,18]. In the present study, however, the specific tissue composition of the individual fractions was not determined. Therefore, the higher antioxidant activity of SBPF3–SBPF5 can be attributed to their measured phytochemical profile rather than to a specific tissue origin. Although SBPF6 contained less TPC and TFC than SBPF3 and SBPF4, its DPPH and FRAP values remained relatively high, suggesting that antioxidant capacity may also depend on the chemical characteristics of the extracted phenolic compounds and on other reducing constituents present in the matrix.

The results show that grinding followed by granulometric fractionation produced SBP fractions with distinct antioxidant properties, with the intermediate fractions, particularly SBPF4, exhibiting the strongest DPPH radical-scavenging and ferric reducing activities. The TPC and TFC profiles further support the potential of dry fractionation by grinding–sieving for obtaining SBP fractions with distinct phytochemical characteristics. Particle size has also been identified as an important factor influencing the physicochemical and functional properties of plant-derived materials [19]. The resulting fraction-specific profiles may therefore be relevant when selecting SBP fractions for different food applications based on the targeted functional and phytochemical properties.

### 3.6. Correlation and Multivariate Analysis

#### 3.6.1. Pearson Correlation Analysis

**Figure 7:**
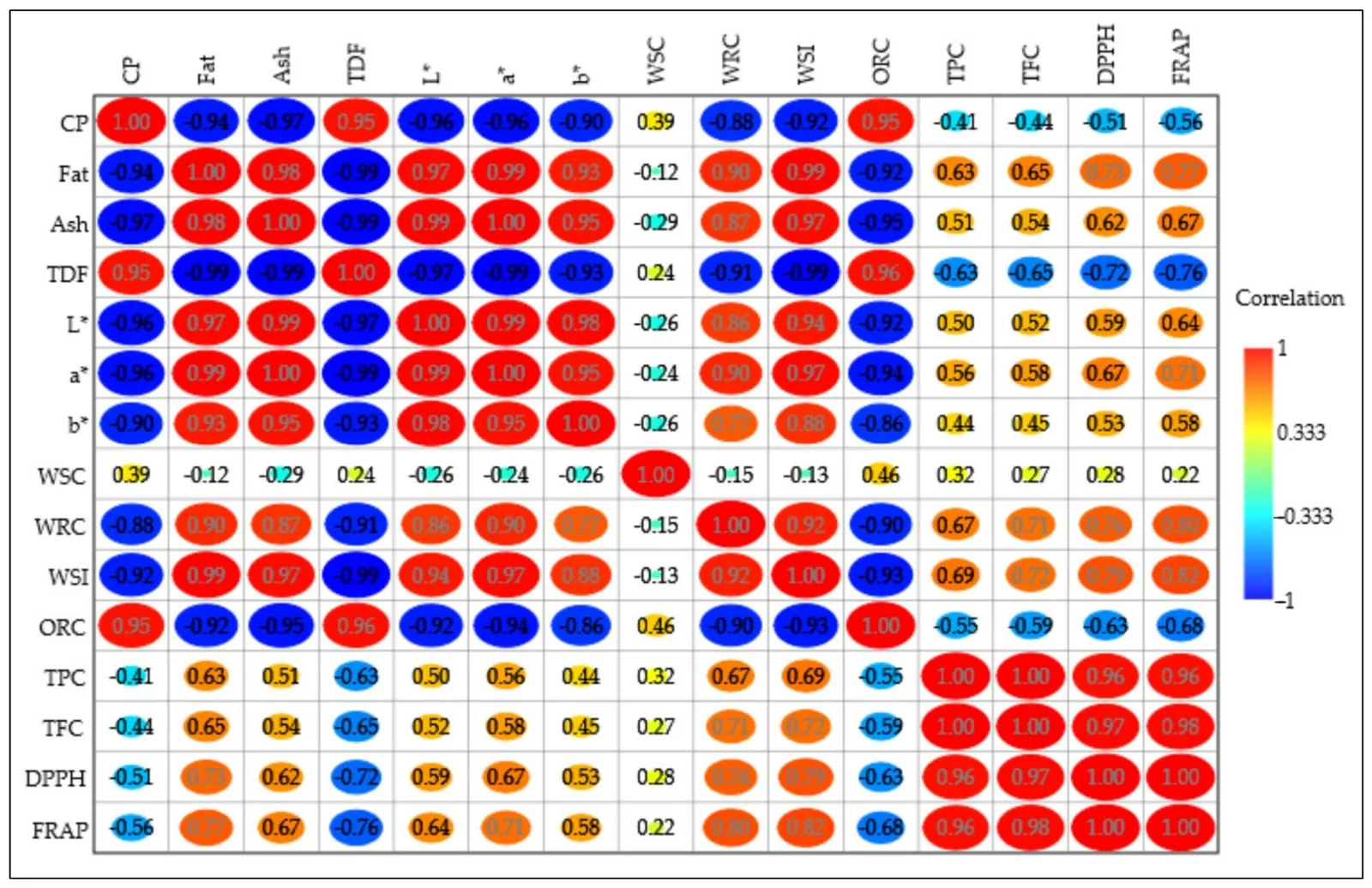
Pearson correlation matrix of compositional, colour, technofunctional, phytochemical, and antioxidant parameters of seed-free sea buckthorn pomace and its granulometric fractions. Values indicate Pearson’s correlation coefficients (r); positive and negative correlations are shown in blue and red, respectively, with the intensity of the shading reflecting correlation strength.

Figure 7 illustrates the Pearson correlation matrix among the compositional (crude protein—CP, fat, ash, and total dietary fibre—TDF), colour ($L^{*}$, $a^{*}$, $b^{*}$), technofunctional (WSC, WRC, WSI, ORC), phytochemical (TPC, TFC), and antioxidant parameters (DPPH, FRAP) of SBP and its granulometric fractions (SBPF1–SBPF6).

Several correlations reflected the compositional and functional differentiation among the granulometric fractions. TDF was positively associated with crude protein (r = 0.95) and ORC (r = 0.96), whereas negative correlations were recorded with WRC and WSI (r = −0.91 and −0.99, respectively). A high positive correlation was also observed between WRC and WSI (r = 0.92), while both parameters were negatively related to ORC (r = −0.90 and −0.93, respectively). These relationships are consistent with the differences in hydration and oil-retention properties observed among fractions with distinct compositional profiles. The colour parameters were also closely related to the compositional characteristics of the granulometric fractions. $L^{*}$, $a^{*}$ and $b^{*}$ showed positive correlations with fat and ash and negative correlations with TDF, while all colour coordinates were negatively associated with ORC. $L^{*}$ was highly correlated with fat (r = 0.97) and ash (r = 0.99), whereas negative correlations were observed with TDF (r = −0.97) and ORC (r = −0.92). These relationships indicate that the visual differences among the granulometric fractions occurred together with changes in their compositional and functional profiles, further supporting the differentiation produced by granulometric separation. WSC showed only weak to moderate correlations with most of the other compositional, colour, technofunctional, phytochemical, and antioxidant parameters. This is consistent with its distinct, non-progressive variation across the particle-size classes and suggests that swelling capacity did not follow the same pattern as the other technofunctional properties.

The highest correlation coefficients were recorded among TPC, TFC, and the antioxidant parameters. TPC was closely associated with TFC (r = 1.00), DPPH (r = 0.96), and FRAP (r = 0.96). High positive correlations were also found between TFC and DPPH (r = 0.97) and between TFC and FRAP (r = 0.98), while DPPH and FRAP followed an almost identical pattern across the samples (r = 1.00). These relationships are consistent with the experimental results obtained for the granulometric fractions, since SBPF3–SBPF5, particularly SBPF4, combined the highest TPC and TFC values with the greatest DPPH radical-scavenging and ferric reducing activities. Positive relationships between phenolic compounds and antioxidant activity have also been reported for sea buckthorn pomace. Wu et al. [9] found high positive correlations between TPC and antioxidant activity in sea buckthorn pomace extracts (r = 0.916–0.951) and concluded that phenolic compounds contributed substantially to their antioxidant properties. Similar relationships have been reported for particle-size-fractionated plant powders, where phenolic and flavonoid contents were closely correlated and were also associated with antioxidant activity [11].

WRC and WSI were positively related to DPPH (r = 0.76 and 0.79, respectively) and FRAP (r = 0.80 and 0.82, respectively), indicating that fractions with higher water-retention and solubility values also generally exhibited higher antioxidant activity.

The correlation analysis showed that differences in composition among SBP and its granulometric fractions were associated with changes in technofunctional, colour, phytochemical, and antioxidant properties. These relationships were further examined by PCA to evaluate how the individual fractions were positioned according to their physicochemical and functional profiles.

#### 3.6.2. Principal Component Analysis

**Figure 8:**
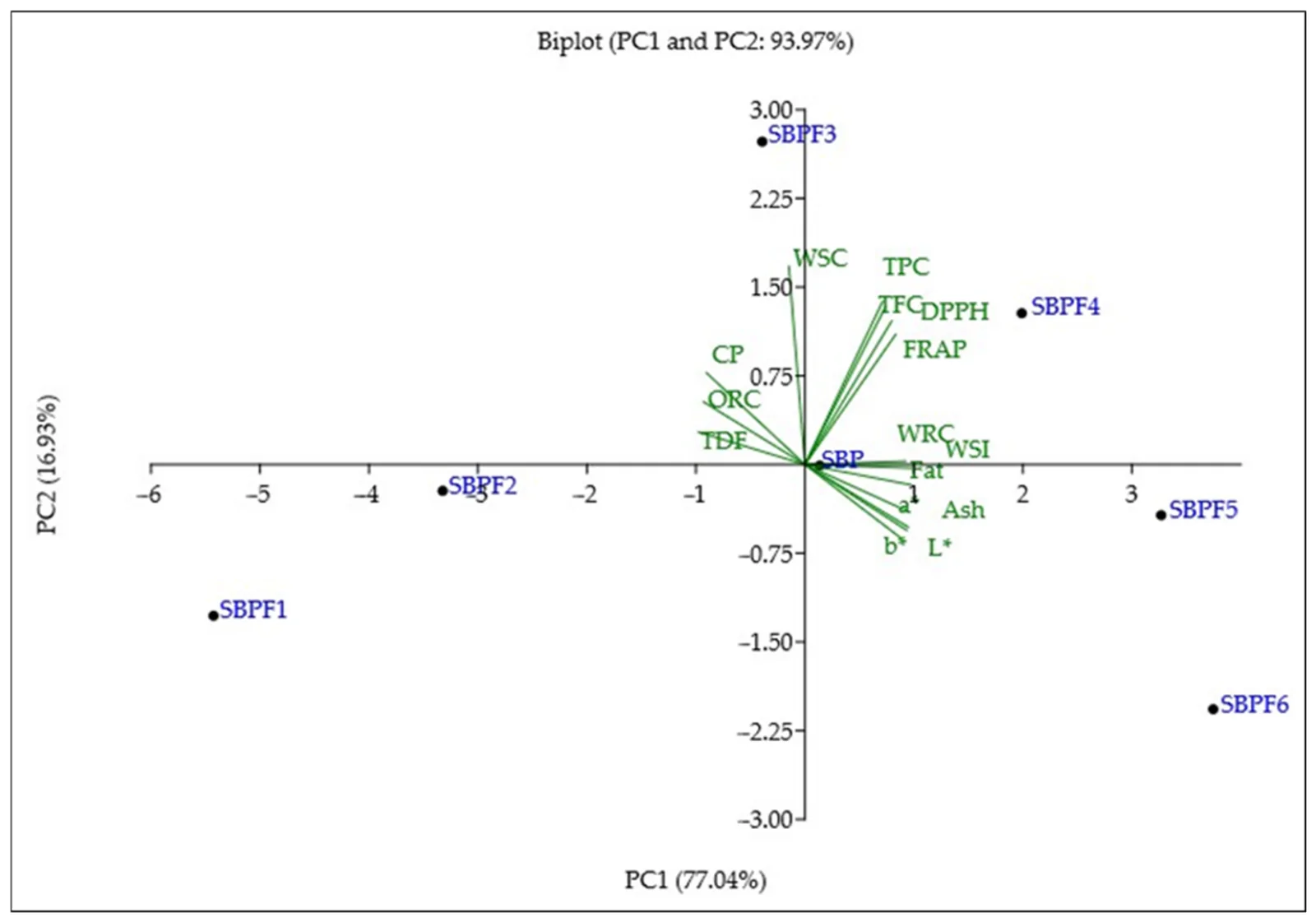
Principal component analysis (PCA) biplot of seed-free sea buckthorn pomace (SBP) and its granulometric fractions (SBPF1–SBPF6). Points represent sample scores, while vectors represent the compositional, colour, technofunctional, phytochemical, and antioxidant variables.

Figure 8 presents the distribution of SBP and its granulometric fractions on the first two principal components. PC1 and PC2 explained 77.04% and 16.93% of the total variance, respectively, accounting for 93.97% of the total variability in the dataset.

PC1 primarily distinguished the variables related to the coarser fractions from those associated with the finer fractions. TDF, crude protein, and ORC were positioned on the negative side of PC1, whereas fat, ash, the colour parameters, WRC, and WSI were positioned on the positive side.

PC2 contributed mainly to the separation of WSC and the phytochemical and antioxidant parameters, with WSC, TPC, TFC, DPPH, and FRAP located in the positive region of this axis. The close orientation of the TPC, TFC, DPPH, and FRAP vectors is consistent with the high positive correlations observed among these variables in the Pearson analysis.

The samples were clearly distributed according to their granulometric and functional profiles. SBPF1 and SBPF2 were positioned on the negative side of PC1, whereas SBPF5 and SBPF6 occupied the positive side, reflecting the compositional and technofunctional differences between the coarser and finer fractions. SBPF3 was mainly separated along PC2 and positioned in the direction of WSC, in agreement with its high swelling capacity. SBPF4 was located in the direction of TPC, TFC, DPPH, and FRAP, corresponding to the high phytochemical content and antioxidant activity measured for this fraction. The unfractionated SBP remained close to the centre of the biplot, indicating a more intermediate profile relative to the separated fractions.

The PCA further illustrated the differentiation among the granulometric fractions based on their combined compositional, technofunctional, colour, phytochemical, and antioxidant profiles. SBPF1 and SBPF2 were associated mainly with fibre-, protein-, and oil-retention-related variables, while SBPF5 and SBPF6 were more closely associated with fat, ash, colour, water retention, and solubility. SBPF3 and SBPF4 showed distinct profiles, associated mainly with WSC and with phytochemical and antioxidant properties, respectively.

#### 3.6.3. Agglomerative Hierarchical Clustering

**Figure 9:**
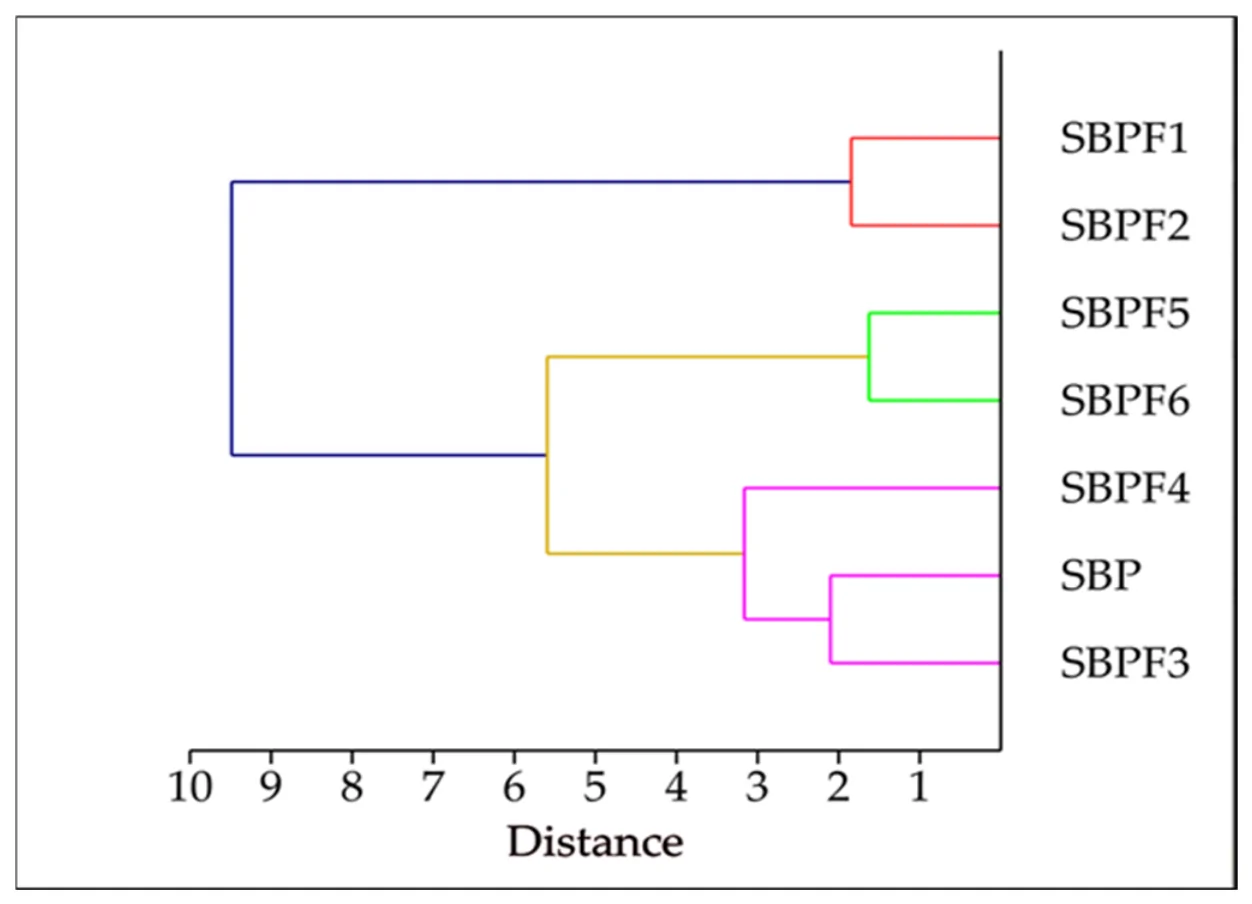
Agglomerative hierarchical clustering (AHC) dendrogram of SBP and its granulometric fractions (SBPF1–SBPF6) based on z-score-standardized compositional, colour, technofunctional, phytochemical, and antioxidant variables, using Ward’s method and Euclidean distance.

Figure 9 presents the hierarchical clustering of SBP and its granulometric fractions based on their multivariate profiles.

Figure 9 presents the agglomerative hierarchical clustering of SBP and its granulometric fractions based on their multivariate profiles. The dendrogram provided a reasonable representation of the original inter-sample distances (cophenetic correlation coefficient = 0.7572). The closest associations were observed between SBPF1 and SBPF2, SBPF5 and SBPF6, and SBP and SBPF3. The coarser fractions SBPF1 and SBPF2 formed a distinct cluster that joined the remaining samples only at the highest linkage distance, indicating marked differences in their multivariate profiles. This grouping is consistent with their comparatively higher TDF, crude protein, and ORC and lower WRC, WSI, and phytochemical values. SBPF5 and SBPF6 also clustered closely, reflecting similarities in their multivariate profiles. SBP clustered with SBPF3, while SBPF4 joined this group at a greater linkage distance. The differentiation of SBPF3 and SBPF4 within this cluster is consistent with the high WSC of SBPF3 and the high TPC, TFC, DPPH, and FRAP values of SBPF4.

The clustering pattern was consistent with the PCA, particularly in the separation of SBPF1–SBPF2 from SBPF5–SBPF6 and in the differentiation of SBPF3 and SBPF4 according to their functional and phytochemical characteristics. Similar multivariate approaches have been used to distinguish plant-derived powders according to particle size and physicochemical characteristics. Ferreira et al. [64] applied hierarchical clustering using Ward’s method and Euclidean distance to differentiate pear pomace samples, while Nabil et al. [63] used PCA to discriminate granulometric fractions of *Opuntia ficus-indica* powder according to their functional, physicochemical, and antioxidant properties.

### 3.7. ATR-FTIR Characterization of SBP and Its Granulometric Fractions

**Figure 10:**
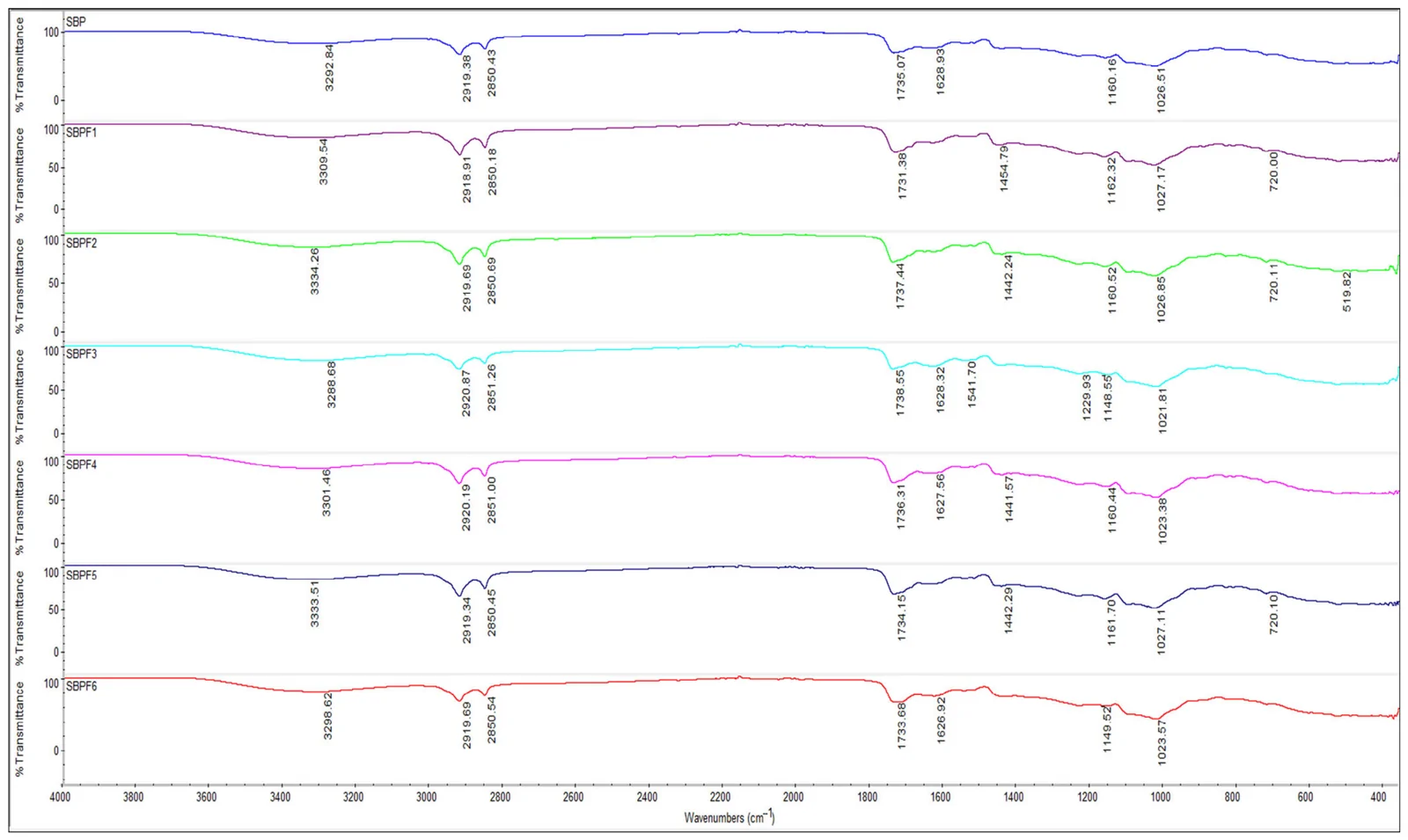
FTIR spectra of sea buckthorn pomace and its granulometric fractions, recorded in the 4000–400 cm^−1^ spectral range (32 scans, 4 cm^−1^ resolution). SBP: unfractionated sea buckthorn pomace; SBPF1–SBPF6: granulometric fractions with particle sizes >2 mm, 1–2 mm, 0.5–1 mm, 0.25–0.5 mm, 0.15–0.25 mm, and 0–0.15 mm, respectively.

The ATR-FTIR spectra of SBP and its granulometric fractions are shown in Figure 10.

All samples exhibited comparable spectral profiles, with the main absorption regions occurring at approximately 3300–3400, 2920–2850, 1730–1740, 1600–1500, and 1200–900 cm^−1^. These regions are commonly associated with O–H stretching, aliphatic C–H stretching, ester carbonyl groups, aromatic/carboxylate vibrations, and carbohydrate-related C–O and C–O–C vibrations, respectively [57,84,85,86,87]. The persistence of these characteristic regions across SBP and SBPF1–SBPF6 indicates that the major chemical functionalities of the pomace remained detectable after grinding and sieving, with no newly appearing absorption bands observed among the granulometric fractions. Comparable spectral profiles characterized by conserved major absorption regions have also been reported for *Hippophae rhamnoides* and other plant-derived matrices [88,89,90].

The broad band located at approximately 3289–3334 cm^−1^ was associated mainly with O–H stretching of hydroxyl-containing constituents, including polysaccharides and phenolic compounds [91,92]. The bands near 2920 and 2850 cm^−1^ corresponded to asymmetric and symmetric stretching of aliphatic C–H groups [92,93], whereas the band at approximately 1731–1739 cm^−1^ was attributed to C=O stretching of esterified groups associated with pectic and lipid-derived constituents [57]. Bands occurring around 1627–1632 cm^−1^ may include contributions from aromatic and carboxylate-containing structures [86,87], while the intense region around 1022–1027 cm^−1^ is characteristic of C–O and C–O–C vibrations associated predominantly with carbohydrate structures [85,87,94].

Although the principal absorption regions were conserved, differences were observed in peak position, intensity, and integrated band area among SBP and its granulometric fractions (Table 5).

To complement the comparison of the spectral parameters presented in Table 5, selected peak-area ratios were calculated from the integrated band areas. The A3300/A2920, A1735/A2920, and A1026/A2920 ratios were considered to compare the relative contributions of hydroxyl-associated, ester/carbonyl-associated, and carbohydrate-associated spectral regions, respectively, relative to the aliphatic C–H region (Table 6).

The peak-area ratios revealed clear differences in the relative spectral contributions of the selected regions among SBP and its granulometric fractions. The A3300/A2920 ratio ranged from 4.11 in SBPF1 to 6.75 in SBPF3. Compared with SBP (5.28), higher values were observed for SBPF3 (6.75) and SBPF6 (6.32), whereas SBPF1, SBPF2, SBPF4, and SBPF5 showed lower ratios. This indicates a comparatively greater contribution of the hydroxyl-associated region relative to the aliphatic C–H region in SBPF3 and SBPF6. The A1735/A2920 ratio showed less pronounced variation, ranging from 1.15 in SBP to 1.52 in SBPF6. All granulometric fractions exhibited values higher than SBP, with the highest ratios recorded for SBPF6 (1.52) and SBPF5 (1.40), indicating a greater relative contribution of ester/carbonyl-associated vibrations in these fractions. The A1026/A2920 ratio also varied markedly among the samples, ranging from 0.40 in SBPF5 to 1.51 in SBPF6. SBPF6 (1.51) and SBPF3 (1.49) showed ratios higher than that of SBP (1.22), whereas lower values were recorded for SBPF2 (1.07), SBPF1 (0.94), SBPF4 (0.47), and SBPF5 (0.40). Thus, the carbohydrate-associated fingerprint region showed a greater relative contribution in SBPF6 and SBPF3 and a lower relative contribution, particularly in SBPF4 and SBPF5, when normalized to the aliphatic C–H region. The three peak-area ratios did not vary in parallel across the particle-size classes. SBPF3 exhibited the highest A3300/A2920 ratio and the second-highest A1026/A2920 ratio, whereas SBPF6 showed relatively high A3300/A2920 and the highest A1735/A2920 and A1026/A2920 ratios. In contrast, SBPF4 and particularly SBPF5 were characterized by low A1026/A2920 ratios despite intermediate A3300/A2920 values and comparatively high A1735/A2920 ratios. This pattern indicates that granulometric separation generated fractions with distinct relative spectral profiles rather than a uniform change in all selected FTIR regions with decreasing particle size.

The variation in peak-area ratios complements the differences observed in the individual spectral parameters and provides a relative comparison of selected chemical regions among the fractions. Because FTIR bands in complex berry-derived matrices may contain overlapping contributions from several constituents [95], these ratios were interpreted as relative spectral indices rather than as direct measures of individual compounds. The use of relative band ratios to distinguish compositional differences while retaining common FTIR fingerprints has also been reported for berry matrices [90]. Similarly, variations in FTIR bands associated with O–H, C–H, C=O, and polysaccharide-related regions have been reported following micronization of raspberry pomace without the appearance of new functional groups [96].

The ATR-FTIR results therefore show that granulometric fractionation was associated with differences in selected spectral parameters and peak-area ratios while preserving the main absorption regions of SBP. The observed variations reflect differences in the relative spectral contributions of hydroxyl-, ester/carbonyl-, and carbohydrate-associated regions among the fractions, whereas no new absorption bands indicative of newly formed chemical functionalities were observed.

### 3.8. Wide-Angle X-Ray Scattering (WAXS) Analysis of SBP and Its Granulometric Fractions

**Figure 11:**
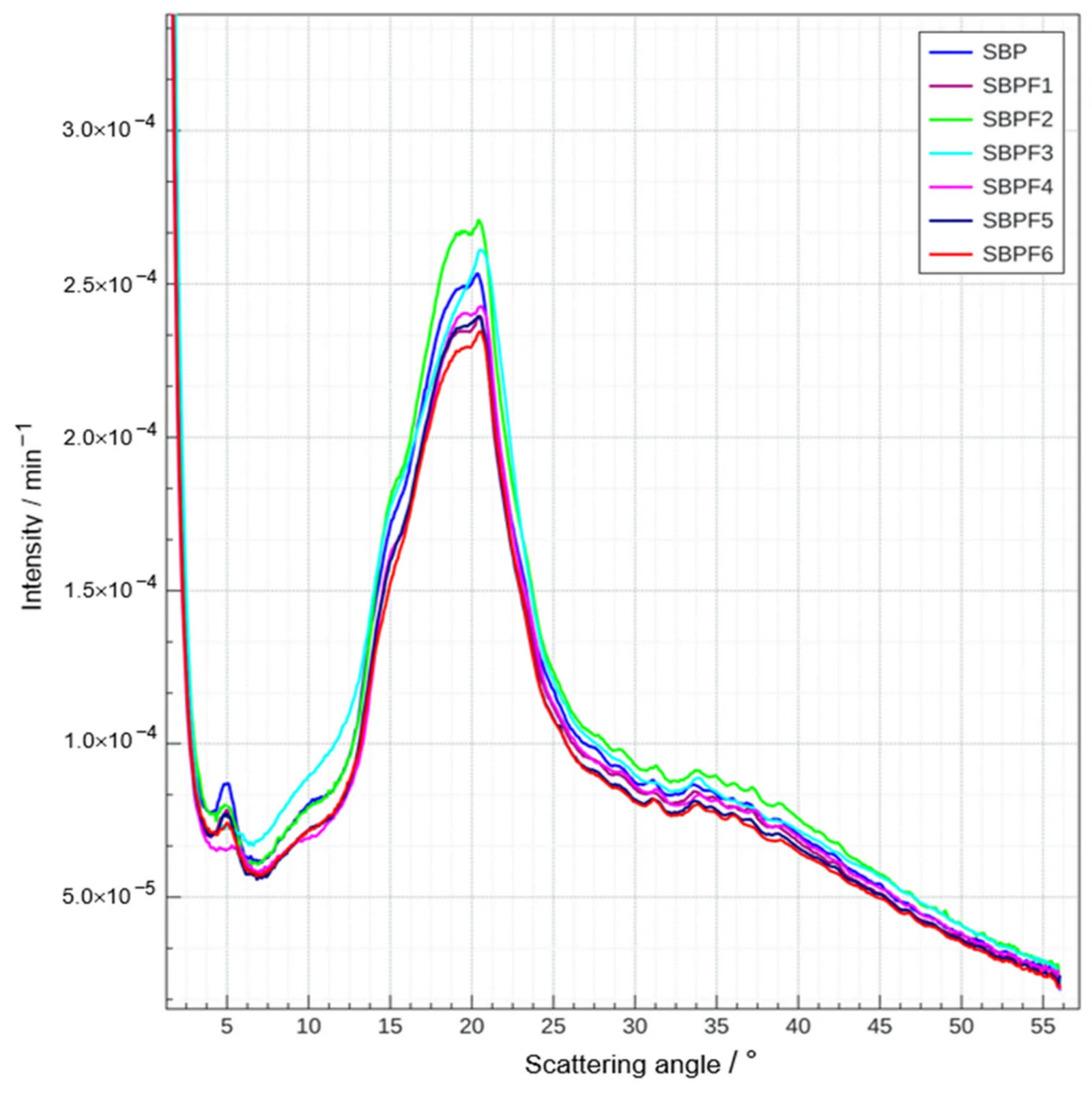
Wide-angle X-ray scattering (WAXS) profiles of SBP and SBPF1–SBPF6. SBP: seed-free sea buckthorn pomace; SBPF1–SBPF6: granulometric fractions with particle sizes >2 mm, 1–2 mm, 0.5–1 mm, 0.25–0.5 mm, 0.15–0.25 mm, and 0–0.15 mm, respectively.

The WAXS profiles of SBP and its granulometric fractions are presented in Figure 11.

All samples exhibited broadly similar scattering patterns dominated by a diffuse maximum centered at a scattering angle (2θ) of approximately 20°, without sharp, well-defined reflections. Such broad scattering features indicate limited long-range structural order and are consistent with the heterogeneous nature of plant-derived and lignocellulosic matrices [57,58,97,98].

The diffuse character of the scattering profiles is consistent with the heterogeneous composition of fruit-derived pomace, which contains cell-wall components with different degrees of structural order. Hemicelluloses, pectic substances, and lignin are predominantly amorphous, whereas cellulose may contain both ordered and less ordered domains [97,98,99,100]. Similar broad scattering profiles have been reported for fruit pomaces and other fibre-rich plant residues, in which the coexistence of several lignocellulosic constituents results in limited long-range ordering [57,101]. Broad amorphous scattering features in the 15–20° region have also been reported for plant-derived protein systems, including globulin, prolamin, and glutelin, whose X-ray diffraction patterns were characterized by limited structural ordering [102].

In cellulose-containing plant materials, characteristic diffraction features are generally associated with ordered cellulose domains. For example, Taharuddin et al. [97] reported diffraction peaks at 14.55°, 22.17°, and 32.36° (2θ) for dragon fruit peel, with the reflection at approximately 22° assigned to the (200) plane of cellulose I. Similarly, cellulose isolated from different fruit and vegetable pomaces exhibited measurable crystalline organization, although the degree of crystallinity varied among botanical sources [98]. However, these studies investigated cellulose-rich or isolated cellulose materials, whereas the present samples represent the whole seed-free pomace matrix containing several cell-wall constituents.

To further compare the scattering profiles, the 2θ positions identified for SBP and the SBPF1–SBPF6 fractions are presented in Table 7.

The SBP showed scattering features at 19.04° and 20.36° within the principal broad region, whereas the corresponding main positions for SBPF1–SBPF6 were located within a narrow range of approximately 20.40–20.50°.

The preservation of this principal scattering region across all fractions (SBPF1–SBPF6) indicates that granulometric fractionation did not produce a substantial displacement of the dominant WAXS feature.

The position of the dominant broad feature in the present samples, around 20° rather than as a sharp reflection near 22°, therefore does not support its direct assignment to a single well-resolved cellulose I reflection. In complex plant cell walls, WAXS signals from crystalline cellulose coexist with broader scattering contributions from other structural components. Del Mundo et al. [99], for example, resolved cellulose I β reflections together with broad scattering contributions associated with pectin and other cell-wall components. Accordingly, the broad maximum observed for SBP and SBPF1–SBPF6 is more appropriately interpreted as a composite feature of the heterogeneous pomace matrix. In addition to the principal region around 20°, several secondary scattering positions were identified at lower and higher 2θ values, with some variation among the individual granulometric fractions (Table 7). However, these secondary features were not consistently present across all samples and were not accompanied by the appearance of new sharp reflections. Thus, although Table 7 reveals fraction-specific differences in individual scattering positions, the scattering pattern remained broadly comparable among SBP and SBPF1–SBPF6. To complement the comparison based on scattering-angle positions, the average crystallite size was estimated from diffraction-peak broadening using the Scherrer method [62], and the resulting values are presented in Table 8.

The average crystallite size varied among SBP and its granulometric fractions, with the lowest value observed for SBPF2 and the highest for SBPF6. Although no consistent trend was evident across the entire particle-size range, a progressive increase was observed from SBPF3 to SBPF6. The observed values indicate differences in crystallite dimensions among the granulometric fractions. The increase in crystallite size towards the finer fractions occurred without a substantial displacement of the principal scattering region around 20° (2θ). This suggests that the fractions differed in the dimensions of the ordered domains contributing to the WAXS signal, rather than showing evidence of a distinct crystalline structural pattern.

Differences in scattering intensity were observed among the samples. Around the principal broad maximum, SBPF2 exhibited the highest intensity, whereas SBPF5 and SBPF6 showed comparatively lower intensities, with the remaining fractions displaying intermediate profiles. SBPF2 showed the lowest crystallite size, whereas SBPF6 exhibited the largest crystallite size despite its lower scattering intensity. Thus, scattering intensity and crystallite size did not vary in parallel across the granulometric fractions.

Changes in diffraction intensity without major shifts in peak position have also been reported for plant-derived materials following processing or changes in sample condition [99,103]. In the present study, the principal scattering region remained around 20° (2θ), while differences were observed in scattering intensity, individual 2θ peak positions, and crystallite size. The increase in crystallite size from SBPF3 to SBPF6 therefore represents a further structural distinction among the finer granulometric fractions.

## 4. Conclusions

The results of this study demonstrate that granulometric fractionation through controlled grinding and sieving is an effective approach for obtaining seed-free sea buckthorn pomace fractions with distinct compositional, phytochemical, technofunctional, colour, and structural characteristics. The coarser fractions were associated with higher total and insoluble dietary fibre contents and greater oil retention capacity, whereas decreasing particle size was accompanied by an increasing contribution of soluble dietary fibre and a declining IDF/SDF ratio, together with higher water solubility. Hydration behaviour varied among particle-size classes, with water swelling capacity and water retention capacity reaching their highest values in different fractions, while the water solubility index increased towards the finer particles. Colour characteristics also changed gradually with decreasing particle size, although the relatively low colour differences indicated that the characteristic appearance of SBP was largely preserved. Pearson correlation analysis revealed clear relationships among several measured properties, particularly strong associations among phenolic content, flavonoid content, DPPH, and FRAP. PCA and hierarchical clustering separated the coarser and finer fractions and highlighted the distinct profiles of SBPF3 and SBPF4. ATR-FTIR analysis showed preservation of the main absorption regions together with fraction-specific differences in spectral parameters and selected peak-area ratios. WAXS indicated broadly comparable scattering profiles, with differences in intensity and secondary scattering features but without the appearance of new sharp reflections; crystallite-size analysis further revealed differences among the fractions, with an increase from SBPF3 to SBPF6. These analyses showed fraction-specific structural differences while the principal spectral and scattering characteristics of SBP remained largely preserved. The novelty of this study lies in the dry fractionation of SBP and the comprehensive characterization of the resulting particle-size classes, revealing distinct compositional, technofunctional, and structural profiles that support a more targeted approach to its valorisation. Granulometric classification may therefore provide a practical basis for tailoring the selection of individual fractions according to the requirements of subsequent food formulations. Further work will focus on extending the characterization of the granulometric fractions through profiling of individual phenolic and carotenoid compounds and a more in-depth investigation of their structural and morphological characteristics, followed by application of selected fractions in relevant food matrices.

## Figures and Tables

**Table 1:** Particle-size distribution and granulometric characteristics of seed-free sea buckthorn pomace fractions obtained by grinding and successive sieving.

Sieve Opening $l_{i}$ (mm)	Average Particle Size of the Fraction $d_{i}$ (mm)	Mass of the Material Retained on the Sieve (g)	Percentage of Material Within Each Particle-Size Class $a_{i}$ (%)	Cumulative Percentage of Material with Particle Size Smaller Than the Sieve Opening $l_{i}$ $T_{i}$ (%)	Cumulative Percentage of Material with Particle Size Larger than the SieveOpening $l_{i}$ $R_{i}$ (%)
0	0.075	10.52 ± 0.31	10.52 ± 0.31	0	100
0.15	0.20	11.96 ± 0.47	11.96 ± 0.47	10.52 ± 0.28	89.48 ± 0.31
0.25	0.375	24.27 ± 0.48	24.27 ± 0.48	22.48 ± 0.31	77.52 ± 0.31
0.50	0.750	26.09 ± 0.53	26.09 ± 0.53	46.75 ± 0.48	53.25 ± 0.48
1.00	1.50	17.81 ± 0.32	17.81 ± 0.32	72.85 ± 0.18	27.15 ± 0.18
2.00	2.414	9.35 ± 0.42	9.35 ± 0.42	90.66 ± 0.42	9.34 ± 0.42

Values are expressed as mean ± standard deviation (SD) of three independent dry fractionation experiments (*n* = 3).

**Table 2:** Proximate composition and energy value of seed-free sea buckthorn pomace and its granulometric fractions.

Parameter	Samples						
	SBP	SBPF1	SBPF2	SBPF3	SBPF4	SBPF5	SBPF6
Moisture (%)	4.91 ± 0.03 ^c^	4.64 ± 0.04 ^d^	5.15 ± 0.02 ^b^	4.95 ± 0.03 ^c^	5.49 ± 0.02 ^a^	4.23 ± 0.03 ^e^	4.12 ± 0.02 ^f^
CP (%)	6.15 ± 0.04 ^c^	6.48 ± 0.03 ^a^	6.42 ± 0.04 ^a^	6.29 ± 0.02 ^b^	6.11 ± 0.03 ^c^	5.72 ± 0.05 ^d^	5.59 ± 0.02 ^e^
Fat (%)	8.25 ± 0.04 ^d^	6.82 ± 0.03 ^g^	7.36 ± 0.02 ^f^	8.11 ± 0.04 ^e^	8.42 ± 0.02 ^c^	8.84 ± 0.05 ^b^	9.35 ± 0.06 ^a^
Ash (%)	2.56 ± 0.03 ^c^	2.33 ± 0.03 ^f^	2.41 ± 0.01 ^e^	2.48 ± 0.03 ^d^	2.59 ± 0.01 ^c^	2.67 ± 0.03 ^b^	2.78 ± 0.02 ^a^
TDF (%)	49.61 ± 0.15 ^d^	58.46 ± 0.17 ^a^	55.37 ± 0.26 ^b^	51.69 ± 0.19 ^c^	47.03 ± 0.14 ^e^	44.81 ± 0.21 ^f^	42.19 ± 0.17 ^g^
IDF (%)	39.82 ± 0.08 ^d^	51.25 ± 0.14 ^a^	47.59 ± 0.12 ^b^	43.18 ± 0.10 ^c^	37.89 ± 0.09 ^e^	34.72 ± 0.13 ^f^	31.16 ± 0.10 ^g^
SDF (%)	9.79 ± 0.09 ^c^	7.21 ± 0.07 ^g^	7.78 ± 0.05 ^f^	8.51 ± 0.08 ^e^	9.14 ± 0.10 ^d^	10.09 ± 0.06 ^b^	11.03 ± 0.09 ^a^
IDF/SDF	4.07 ± 0.10 ^d^	7.11 ± 0.12 ^a^	6.12 ± 0.07 ^b^	5.07 ± 0.05 ^c^	4.15 ± 0.08 ^d^	3.44 ± 0.05 ^e^	2.83 ± 0.06 ^f^
CRB	28.52 ± 0.14 ^d^	21.27 ± 0.11 ^g^	23.29 ± 0.09 ^f^	26.48 ± 0.12 ^e^	30.36 ± 0.14 ^c^	33.73 ± 0.10 ^b^	35.97 ± 0.13 ^a^
Energy Value (kcal/100 g)	312.15 ± 0.31 ^d^	289.30 ± 0.29 ^g^	295.82 ± 0.26 ^f^	307.45 ± 0.36 ^e^	315.72 ± 0.42 ^c^	326.98 ± 0.25 ^b^	334.77 ± 0.34 ^a^

CP: crude protein; TDF: total dietary fibre; IDF: insoluble dietary fibre; SDF: soluble dietary fibre; CRB: available carbohydrates; SBP: unfractionated seed-free sea buckthorn pomace; SBPF1–SBPF6: granulometric fractions with particle sizes of >2 mm, 1–2 mm, 0.5–1 mm, 0.25–0.5 mm, 0.15–0.25 mm, and 0–0.15 mm, respectively. Values are expressed as mean ± standard deviation (SD) of three independent experimental replicates (*n* = 3). Different superscript letters (a–g) within the same column indicate statistically significant differences among samples (*p* < 0.05, one-way ANOVA).

**Table 3:** Hydration and oil retention properties of seed-free sea buckthorn pomace and its granulometric fractions.

Technofunctional Properties	Samples						
	SBP	SBPF1	SBPF2	SBPF3	SBPF4	SBPF5	SBPF6
WSC (mL/g)	2.65 ± 0.03 ^b^	1.98 ± 0.02 ^f^	2.54 ± 0.03 ^c^	3.51 ± 0.04 ^a^	2.16 ± 0.02 ^d^	2.05 ± 0.03 ^e^	2.01 ± 0.038 ^e^
WRC (g/g)	1.87 ± 0.02 ^e^	1.28 ± 0.05 ^g^	1.71 ± 0.02 ^f^	1.94 ± 0.03 ^d^	2.13 ± 0.05 ^c^	2.68 ± 0.04 ^a^	2.31 ± 0.03 ^b^
WSI (%)	9.79 ± 0.05 ^e^	7.03 ± 0.07 ^g^	8.31 ± 0.08 ^f^	10.08 ± 0.10 ^d^	11.13 ± 0.06 ^c^	11.75 ± 0.08 ^b^	12.59 ± 0.07 ^a^
ORC (g/g)	2.39 ± 0.05 ^c^	2.96 ± 0.04 ^a^	2.91 ± 0.03 ^a^	2.68 ± 0.06 ^b^	1.80 ± 0.04 ^d^	1.47 ± 0.05 ^e^	1.42 ± 0.03 ^e^

SBP: unfractionated seed-free sea buckthorn pomace; SBPF1–SBPF6: granulometric fractions with particle sizes of >2 mm, 1–2 mm, 0.5–1 mm, 0.25–0.5 mm, 0.15–0.25 mm, and 0–0.15 mm, respectively. Values are expressed as mean ± standard deviation (SD) of three independent experimental replicates (*n* = 3). Different superscript letters (a–g) within the same row indicate statistically significant differences among samples (*p* < 0.05, one-way ANOVA).

**Table 4:** Analysis of colour properties of seed-free sea buckthorn pomace and its granulometric fractions.

Colour Measurements	Samples						
	SBP	SBPF1	SBPF2	SBPF3	SBPF4	SBPF5	SBPF6
$L^{*}$	52.41 ± 0.03 ^c^	51.13 ± 0.04 ^g^	51.49 ± 0.02 ^f^	51.78 ± 0.04 ^e^	52.22 ± 0.03 ^d^	52.73 ± 0.02 ^b^	53.15 ± 0.06 ^a^
$a^{*}$	17.63 ± 0.05 ^c^	16.81 ± 0.02 ^f^	17.16 ± 0.03 ^e^	17.38 ± 0.04 ^d^	17.71 ± 0.05 ^c^	18.01 ± 0.03 ^b^	18.27 ± 0.04 ^a^
$b^{*}$	45.06 ± 0.02 ^b^	43.62 ± 0.06 ^g^	43.96 ± 0.05 ^f^	44.12 ± 0.03 ^e^	44.63 ± 0.02 ^d^	44.98 ± 0.02 ^c^	45.51 ± 0.06 ^a^
C	48.38 ± 0.04 ^b^	46.75 ± 0.03 ^f^	47.19 ± 0.02 ^e^	47.42 ± 0.03 ^d^	48.02 ± 0.06 ^c^	48.45 ± 0.04 ^b^	49.04 ± 0.05 ^a^
TCI	71.33 ± 0.04 ^c^	69.28 ± 0.03 ^g^	69.84 ± 0.05 ^f^	70.21 ± 0.03 ^e^	70.94 ± 0.04 ^d^	71.61 ± 0.04 ^b^	72.32 ± 0.03 ^a^
$h^{{}^{\circ}}$	68.63 ± 0.07 ^b^	68.92 ± 0.05 ^a^	68.68 ± 0.04 ^b^	68.50 ± 0.03 ^c^	68.36 ± 0.05 ^d^	68.18 ± 0.02 ^e^	68.13 ± 0.02 ^f^
∆E	-	2.09 ± 0.04 ^a^	1.51 ± 0.03 ^b^	1.16 ± 0.05 ^c^	0.47 ± 0.02 ^d^	0.50 ± 0.02 ^d^	1.08 ± 0.04 ^c^

SBP: unfractionated seed-free sea buckthorn pomace; SBPF1–SBPF6: granulometric fractions with particle sizes of >2 mm, 1–2 mm, 0.5–1 mm, 0.25–0.5 mm, 0.15–0.25 mm, and 0–0.15 mm, respectively. $L^{*}$: lightness; $a^{*}$: red–green coordinate; $b^{*}$: yellow–blue coordinate; ΔE: total colour difference compared with the seed-free sea buckthorn pomace (control); C: chroma; $h^{{}^{\circ}}$: hue angle; TCI: total colour intensity. Values are expressed as mean ± standard deviation (SD) of three independent experimental replicates (*n* = 3). Different superscript letters (a–g) within the same row indicate statisticant differences among samples (p < 0.05, one-way ANOVA).

**Table 5:** Peak positions, intensities, and integrated band areas of selected ATR-FTIR bands of seed-free sea buckthorn pomace (SBP) and its granulometric fractions (SBPF1–SBPF6).

Spectrum: SBP			Spectrum: SBPF1			Spectrum: SBPF2			Spectrum: SBPF3		
Absolute threshold: 96.711			Absolute threshold: 93.322			Absolute threshold: 95.602			Absolute threshold: 94.257		
Sensitivity: 50			Sensitivity: 50			Sensitivity: 50			Sensitivity: 50		
Peak list:			Peak list:			Peak list:			Peak list:		
Position (cm^−1^)	Intensity (T, %)	Area	Position (cm^−1^)	Intensity (T, %)	Area	Position (cm^−1^)	Intensity (T, %)	Area	Position (cm^−1^)	Intensity (T, %)	Area
–	–	–	–	–	–	519.82	60.820	30.75	–	–	–
–	–	–	720.00	67.425	52.65	720.11	70.949	45.99	–	–	–
1026.51	47.661	981.84	1027.17	51.100	845.08	1026.85	56.147	814.82	1021.81	52.570	896.30
1160.16	60.882	145.45	1162.32	61.283	194.65	1160.52	65.883	165.20	1148.55	66.479	73.20
–	–	–	1454.79	75.920	196.51	1442.24	78.788	49.29	1229.93	67.874	208.08
–	–	–	–	–	–	–	–	–	1541.70	82.610	30.05
1628.93	74.875	105.06	1629.71	78.240	104.85	1631.74	81.430	83.40	1628.32	76.069	353.74
1735.07	67.976	928.67	1731.38	66.945	1192.56	1737.44	70.832	1036.37	1738.55	73.115	735.70
2850.43	73.757	201.05	2850.18	72.157	263.26	2850.69	76.148	220.60	2851.26	79.498	159.84
2919.38	65.345	805.12	2918.91	63.530	896.30	2919.69	68.371	764.02	2920.87	72.710	603.30
3292.84	81.856	4251.17	3309.54	84.479	3684.50	3334.26	86.274	3183.48	3288.68	82.569	4072.99
**Spectrum: SBPF4**			**Spectrum: SBPF5**			**Spectrum: SBPF6**					
Absolute threshold: 95.990			Absolute threshold: 93.077			Absolute threshold: 90.303					
Sensitivity: 50			Sensitivity: 50			Sensitivity: 50					
Peak list:			Peak list:			Peak list:					
Position (cm^−1^)	Intensity (T, %)	Area	Position (cm^−1^)	Intensity (T, %)	Area	Position (cm^−1^)	Intensity (T, %)	Area			
–	–	–	–	–	–	–	–	–			
–	–	–	720.10	67.379	49.92	–	–	–			
1023.38	51.482	363.44	1027.11	50.509	337.52	1023.57	42.595	1104.08			
1160.44	63.876	149.75	1161.70	61.653	189.49	1149.52	60.639	80.91			
–	–	–	–	–	–	–	–	–			
1441.57	77.321	12.73	1442.29	76.202	11.06	–	–	–			
1627.56	78.899	98.81	1629.71	78.530	81.57	1626.92	74.363	117.12			
1736.31	68.492	1055.08	1734.15	66.929	1175.90	1733.68	65.918	1108.65			
2851.00	75.846	221.93	2850.45	73.278	241.18	2850.54	74.900	198.20			
2920.19	67.854	780.35	2919.34	64.676	841.90	2919.69	67.062	730.08			
3301.46	84.768	3678.55	3333.51	84.164	3830.6	3298.62	80.787	4612.84			

(–) indicates that no corresponding peak was reported in the generated peak list for the respective spectrum under the applied peak-finding settings. SBP: seed-free sea buckthorn pomace; SBPF1–SBPF6: granulometric fractions with particle sizes >2 mm, 1–2 mm, 0.5–1 mm, 0.25–0.5 mm, 0.15–0.25 mm, and 0–0.15 mm, respectively.

**Table 6:** Selected ATR-FTIR peak-area ratios of seed-free sea buckthorn pomace (SBP) and its granulometric fractions (SBPF1–SBPF6).

Peak-Area Ratio	Samples						
	SBP	SBPF1	SBPF2	SBPF3	SBPF4	SBPF5	SBPF6
A3300/A2920	5.28	4.11	4.17	6.75	4.71	4.55	6.32
A1735/A2920	1.15	1.33	1.36	1.22	1.35	1.40	1.52
A1026/A2920	1.22	0.94	1.07	1.49	0.47	0.40	1.51

A: the integrated area of the corresponding ATR-FTIR band. Nominal wavenumbers refer to the corresponding bands identified within each sample. SBP: seed-free sea buckthorn pomace; SBPF1–SBPF6: granulometric fractions with particle sizes >2 mm, 1–2 mm, 0.5–1 mm, 0.25–0.5 mm, 0.15–0.25 mm, and 0–0.15 mm, respectively.

**Table 7:** Scattering angle (2θ) peak positions from WAXS analysis of sea buckthorn pomace (SBP) and its granulometric fractions (SBPF1–SBPF6).

Sample	Scattering Angle, 2θ (°)											
SBP	5.11	–	10.27	15.08	19.04	20.36	29.01	31.18	33.47	34.56	36.97	38.69
SBPF1	5.01	–	–	15.08	19.23	20.49	28.80	30.98	33.56	34.76	37.17	38.58
SBPF2	4.82	–	8.17	15.47	19.33	20.40	29.00	31.28	33.66	34.96	37.26	38.89
SBPF3	5.01	5.97	–	14.98	–	20.50	–	31.28	33.96	36.06	37.17	39.09
SBPF4	5.58	–	9.32	15.27	19.04	20.49	28.09	31.08	33.86	34.76	37.27	42.56
SBPF5	5.01	–	–	15.37	19.04	20.40	29.10	31.08	33.66	34.66	37.17	38.79
SBPF6	5.01	–	–	13.92	19.04	20.49	29.05	31.07	33.56	34.66	37.27	38.99

SBP: unfractionated seed-free sea buckthorn pomace; SBPF1–SBPF6: granulometric fractions with particle sizes of >2 mm, 1–2 mm, 0.5–1 mm, 0.25–0.5 mm, 0.15–0.25 mm, and 0–0.15 mm.

**Table 8:** Crystallite size of sea buckthorn pomace and its granulometric fractions.

Samples	Crystallite Size (Å)
SBP	63.67
SBPF1	70.66
SBPF2	62.00
SBPF3	73.00
SBPF4	75.33
SBPF5	77.33
SBPF6	99.67

SBP: unfractionated seed-free sea buckthorn pomace; SBPF1–SBPF6: granulometric fractions with particle sizes of >2 mm, 1–2 mm, 0.5–1 mm, 0.25–0.5 mm, 0.15–0.25 mm, and 0–0.15 mm.

## Data Availability

The original contributions presented in this study are included in the article. Further inquiries can be directed to the corresponding author.
